# Subcellular stoichiogenomics reveal cell evolution and electrostatic interaction mechanisms in cytoskeleton

**DOI:** 10.1186/s12864-018-4845-0

**Published:** 2018-06-18

**Authors:** Yu-Juan Zhang, Chengxu Zhu, Yiran Ding, Zheng-Wen Yan, Gong-Hua Li, Yang Lan, Jian-Fan Wen, Bin Chen

**Affiliations:** 1 Institute of Entomology and Molecular Biology, College of Life Sciences, Chongqing Normal University, Shapingba, Chongqing, 401331 People’s Republic of China; 2 State Key Laboratory of Genetic Resources and Evolution, Kunming Institute of Zoology, Chinese Academy of Sciences, Kunming, Yunnan Province 650223 People’s Republic of China

**Keywords:** Stoichiogenomics, Bioinformatics, Genomics, Element, Charge amino acid

## Abstract

**Background:**

Eukaryotic cells contain a huge variety of internally specialized subcellular compartments. Stoichiogenomics aims to reveal patterns of elements usage in biological macromolecules. However, the stoichiogenomic characteristics and how they adapt to various subcellular microenvironments are still unknown.

**Results:**

Here we first updated the definition of stoichiogenomics. Then we applied it to subcellular research, and detected distinctive nitrogen content of nuclear and hydrogen, sulfur content of extracellular proteomes. Specially, we found that acidic amino acids (AAs) content of cytoskeletal proteins is the highest. The increased charged AAs are mainly caused by the eukaryotic originated cytoskeletal proteins. Functional subdivision of the cytoskeleton showed that activation, binding/association, and complexes are the three largest functional categories. Electrostatic interaction analysis showed an increased electrostatic interaction between both primary sequences and PPI interfaces of 3D structures, in the cytoskeleton.

**Conclusions:**

This study creates a blueprint of subcellular stoichiogenomic characteristics, and explains that charged AAs of the cytoskeleton increased greatly in evolution, which offer material basis for the eukaryotic cytoskeletal proteins to act in two ways of electrostatic interactions, and further perform their activation, binding/association and complex formation.

**Electronic supplementary material:**

The online version of this article (10.1186/s12864-018-4845-0) contains supplementary material, which is available to authorized users.

## Background

One of the major transitions in cell evolution is the appearance of cell compartmentalization, which makes a distinction between prokaryotes and eukaryotes [1]. Prokaryotic cells have no subcellular compartments, while eukaryotic cells contain a huge variety of internally specialized subcellular compartments like the nucleus, mitochondria, and chloroplast, commonly surrounded by double-layered membranes, and endoplasmic reticulum, Golgi, lysosome, and peroxisome, surrounded by single-layered membranes. Each subcellular location has its own function, structure and physio-chemical environment [2, 3]. As organisms compete for living space, proteins also function in different physio-chemical environments and evolve to adapt to the environments in which they work [4], which in turn influences their primary and higher structures, because homologs, or proteins with high similarity in structure/function, live in the same place [2]. Attempts have been made to reveal the characteristics of proteins in different subcellular locations. It was indicated that extracellular proteins evolved faster than intracellular proteins [5], and that relocated proteins functionally adapted to their new subcellular environments and developed new functional roles after gene duplication [3].

The cytoskeleton is an open system in the cells. Unlike membranes-surrounded compartments, it is distributed extensively throughout the cells to support the cellular shape, movement, intracellular transportation, polarity and division [6]. To achieve these functions, the cytoskeleton integrates a wide variety of proteins, which often form dynamic and adaptive structures, such as microfilaments, intermediate filaments and microtubules [6]. Therefore, the interactions among proteins are most important for the cytoskeleton’s function. Advances in cellular biology and biochemistry have revealed that the net charge plays important roles in the treadmilling phenomenon, which unchanged the net length of the treadmilling filaments (actin filaments or microtubules) by keeping the speed of growth at the (+) end equal to the rate of shrinkage at the (−) end [7, 8]. However, whether the charge affects other cytoskeletal proteins or affects them in a different way remains unknown. In addition, changes in the cytoskeleton are key diagnostic indicators in the pathology of some diseases, including cancers [9]. Therefore, understanding the basic charge characteristics of the cytoskeleton can contribute to understanding, diagnosis, and therapy for various diseases.

Stoichiogenomics is a new field, which aims to reveal patterns of the differential usage of key elements [e.g. oxygen (O)] in proteins and nucleic acids [10, 11]. With the advances in the next-generation sequencing (NGS) and assembly algorithms, more and more genomic, metagenomic and metatranscriptomic data is now available. Stoichiogenomics provides a new way to interpret those omics data [12, 13]. Stoichiogenomics is composed of “Stoichio” and “genomics”. In chemistry, stoichiometry is the calculation of relative quantities of reactants and products in chemical reactions [14]. So in a broad sense, the definition of stoichiogenomics should be to examine quantities and measurable chemical properties of material composition in omics [11]. These materials include elements, monomers, and functional groups of monomers, and even chemical properties that could be measured in nucleotides, proteins and metabolites. Charged amino acid residues are significant contributors to the free energy of binding for protein-protein interactions by creating salt bridges and salt bridge networks, and introducing specificity in binding [15]. Because of their roles in protein functions and structures, charged AAs apparently can be important targets for selective forces in protein evolution [16]. Our laboratory has accumulated relevant research in stoichiogenomics, studying macroevolutionary trends of five elements (oxygen, sulfur, nitrogen, carbon, and hydrogen) and their related functional groups [17]. However, what are the characteristics of elements and charged AA compositions of proteins in different subcellular locations? Whether subcellular environments drive protein evolution with respect to stoichiogenomics is still unknown. The aim of this study is to examine different patterns of stoichiogenomic characteristics of various subcellular proteomes, especially the cytoskeleton, and interpret the functional mechanisms of how charged AAs play roles in the cytoskeleton based on stoichiogenomics.

As the first stoichiogenomics study of subcellular proteomes, this provides an overview of stoichiogenomic characteristics. Among all investigated subcellular proteomes, the cytoskeleton’s acidic AA and the nuclear proteome’s nitrogen contents were the highest, and the extracellular proteome’s hydrogen content was the lowest. Also, the extracellular sulfur content was very high. In evolutionary analysis, we found that eukaryotic proteins have more basic AAs than prokaryotes. The cytoskeleton’s basic AA and means acidic AA contents were higher than their prokaryotic analogs. Most cytoskeletal protein families’ charged AA contents were higher than the mean content of eukaryotic proteins, and some were even two times higher than the average level (e.g. the acidic AA content of Troponins (TnC), Tropomyosins (TPM), Inter IV and Stathmin (Lamin). Functional subdivision results suggested that the cytoskeleton is closely associated with binding, dissociation and building complexes. More electrostatic interactions were detected in both primary sequences and 3D structures, which revealed an integrated functional interpretation of charge in cytoskeleton. This work provides a comprehensive study of stoichiogenomic characteristics of subcellular proteomes, especially charge characteristics, evolutionary law, and functional interpretation of the cytoskeleton. Stoichiogenomics not only provides a way to interpret big data [7, 8], but also provides a better understanding of microenvironment-driven protein evolution, and is relevant for understanding the molecular mechanisms of macromolecular machines. Results obtained here could also be useful for basic cell biology and functional interpretation of the cytoskeleton and contributing to the pathology of cytoskeleton-related disease. Furthermore, this study lays a foundation for applying stoichiogenomics to research of metagenomics and unresolved evolutionary and species classification problems.

## Results

### Stoichiogenomic characteristics of subcellular proteomes

**Fig. 1:**
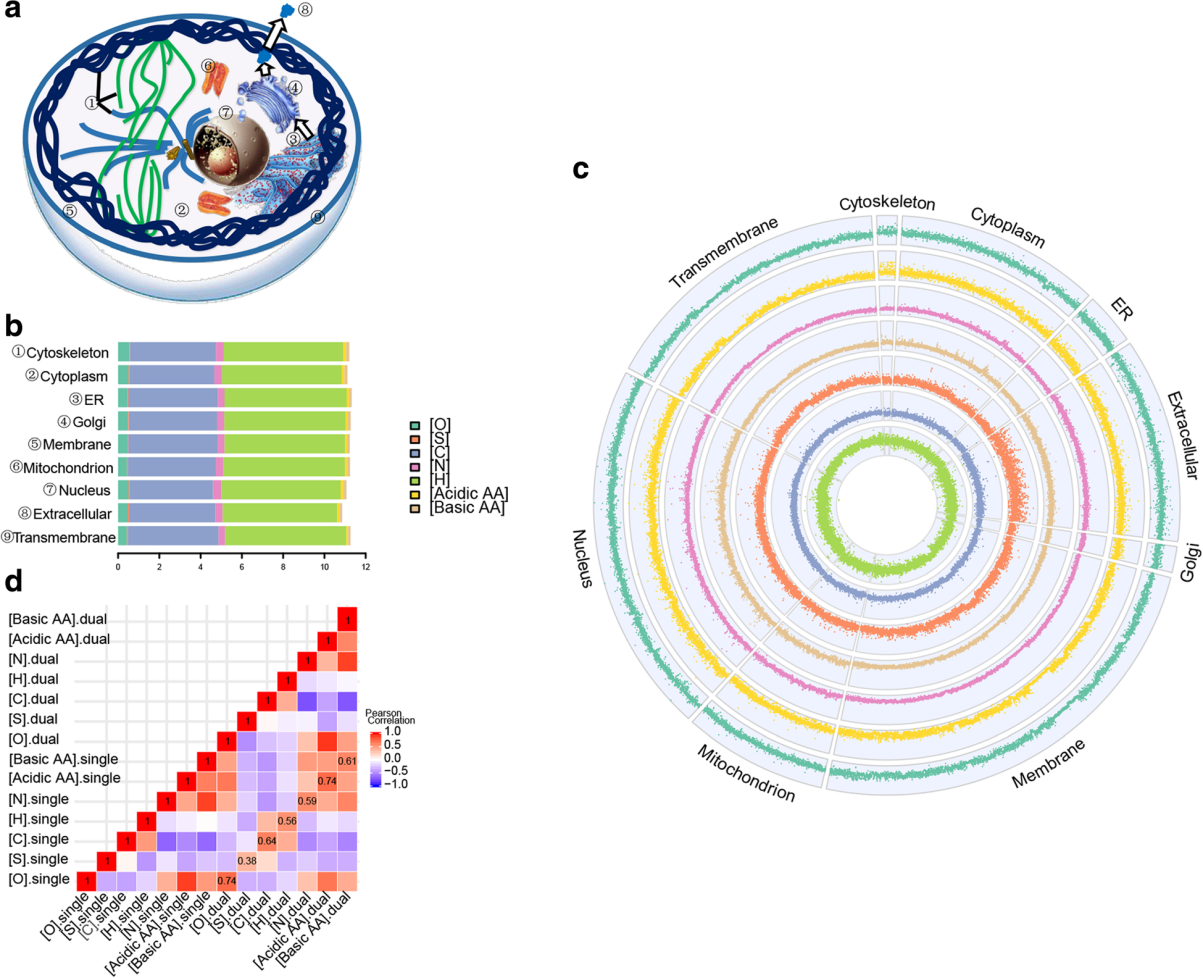
Stoichiogenomic characteristics of subcellular proteomes. **a** The subcellular locations analyzed in this study. For more detail, see Supplementary information, Additional file 1: Tables S1-S2. **b** Histogram showing the mean contents of oxygen, sulfur, nitrogen, carbon, hydrogen, acidic AA and basic AA, calculated from single location proteins. **c** Circle plot showing the distribution of 7 kinds of stoichiogenomic characteristics (as same as the (**b**) of each studied protein from 9 subcellular proteomes and 12 species, calculated from single location proteins. **d** Pairwise correlation analysis of stoichiogenomic characteristics between single and dual location proteins

By filtering sequences retrieved from Swiss-Prot, we obtained single and dual location proteins from 9 subcellular locations (Fig. 1a) and 12 organisms (Additional file 1: Table S1 and S2). Then, we studied 7 kinds of stoichiogenomic characteristics, including five kinds of elements (oxygen, sulfur, nitrogen, carbon, hydrogen) and charged (acidic and basic) AA compositions, of various subcellular proteomes. The mean level of each subcellular proteome’s element and charged AA composition were shown (Fig. 1b).

**Fig. 2:**
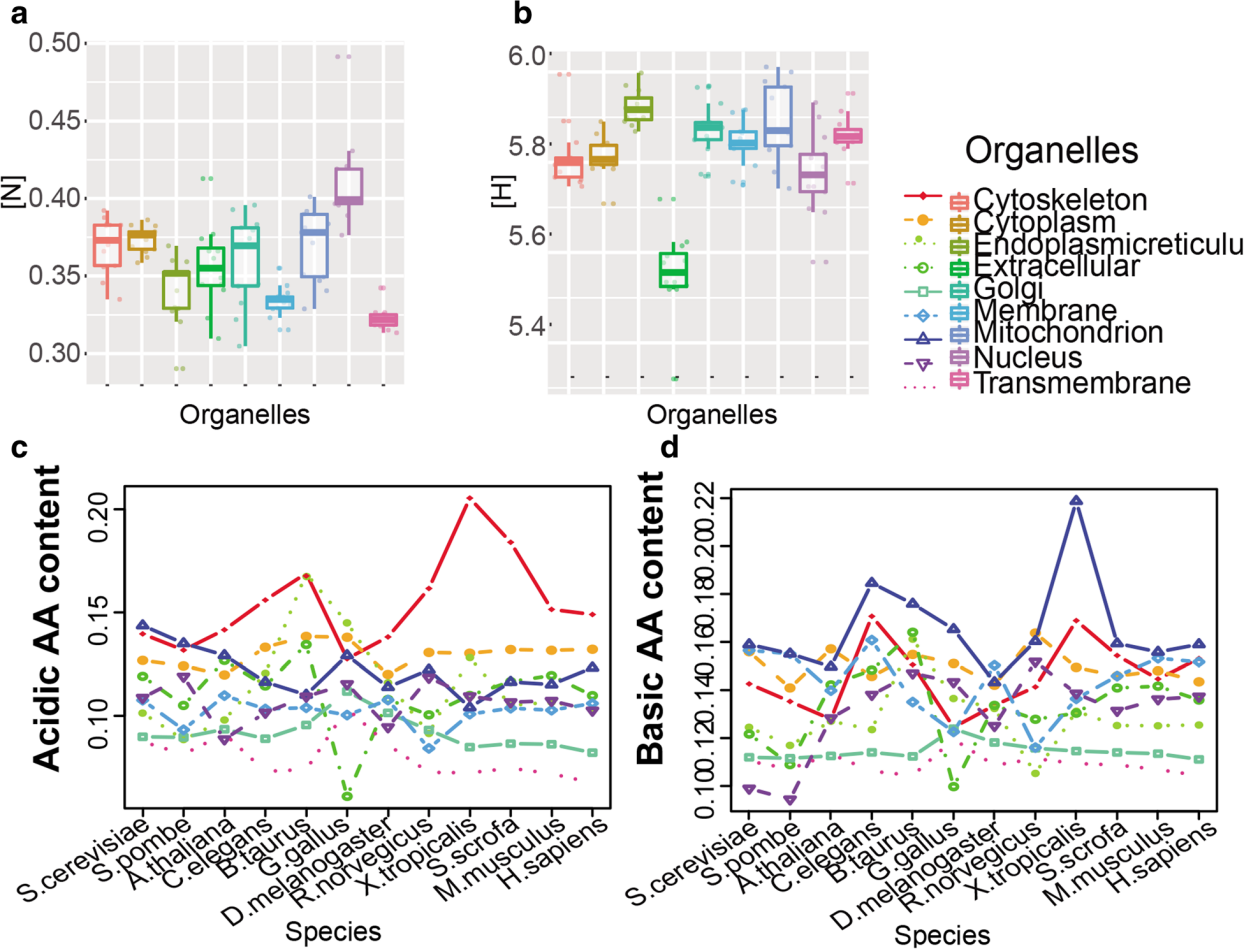
Charged AA, nitrogen and hydrogen characteristics of subcellular proteomes. **a** Boxplot showing the nitrogen contents of various subcellular proteomes calculated from single location proteins. [N]: nitrogen contents. **b** Boxplot showing the hydrogen contents of various subcellular proteomes calculated from single location proteins. [H]: hydrogen contents. **c** The distribution of average acidic AA content of different subcellular proteomes in different species, calculated from single location proteins. **d** The distribution of average basic AA content of different subcellular proteomes in different species, calculated from single location proteins

A circle plot was used to show the overall distribution of subcellular proteome-wide stoichiogenomic characteristics (Fig. 1c). After comparison between groups for each stoichiogenomic characteristics (Additional file 1: Figure S2–8), we detected 3 significant differences (*p* < 0.01). Among all the subcellular proteomes investigated, the nucleus proteome’s nitrogen content was the highest (Fig. 1c, 2A and Additional file 1: Figure S5). The extracellular proteome’s hydrogen content was the lowest, also its sulfur composition was relatively high (Figs. 1c, 2b and Additional file 1: Figure S3, S6).

In addition, high correlations between oxygen and acidic AA contents, and between nitrogen and basic AA contents were detected (Fig. 1d and Additional file 1: Figure S9). These stoichiogenomic characteristics were also calculated for the dual location proteins. Pearson correlation analysis of stoichiogenomic characteristics obtained from single and dual location proteins showed a relatively high correlation value (around 0.6), however that of sulfur is very low (Fig. 1d).

Furthermore, we compared the charged AA contents of the single location proteins with different locations in each species. The results showed that 1) for acidic AA, the mean content of cytoskeletal proteins is higher than that of the other subcellular proteomes in most species, except in *S. pombe* and *C. elegans* (Fig. 2c and Additional file 1: Figure S7). 2) For basic AA, the mean content of the nuclear proteome is higher than most other subcellular proteomes, except in *A. thaliana*. In addition, cytoskeletal proteins have a relatively higher basic AA content (Fig. 2d and Additional file 1: Figure S8). 3) The magnitude of the difference for subcellular proteomes is different in different species. In well-studied model species, such as *Saccharomyces cerevisiae*, *S. pombe*, *A. thaliana*, *D. melanogaster*, *M. musculus* and *H. sapiens*, the contents change little. In the other species, the contents vary greatly among different subcellular locations. 4) Furthermore, we analyzed the acidic and basic AA contents of the dual location proteins to test whether dual subcellular locating characteristic blurs the results we obtained above. The results obtained from dual location proteins (Additional file 1: Figure S10) are almost identical to those of the single location proteins.

### Evolutionary law of the charged AA contents between the proteins of eukaryotic cells and prokaryotic cells

**Fig. 3:**
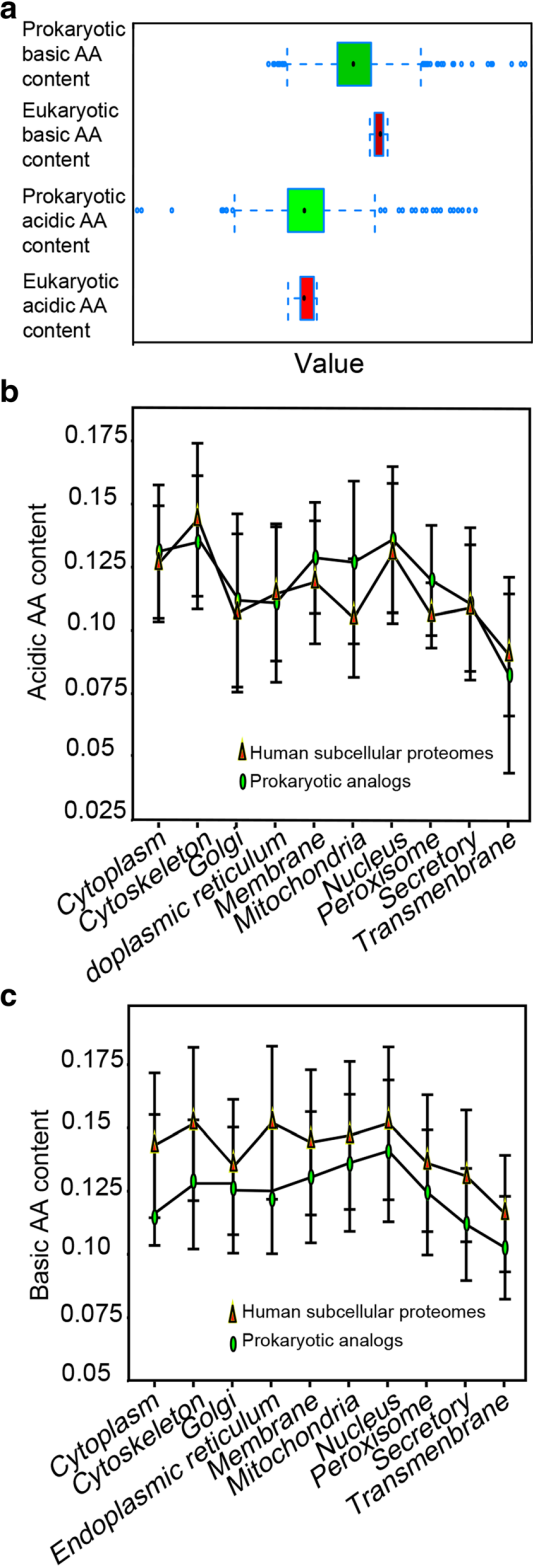
The charged amino acid contents of proteins in eukaryotic cells and prokaryotic cells. **a** The charged amino acid contents of predicted proteins from 66 eukaryotic and 1051 prokaryotic genomes. **b** The acidic AA contents of eukaryotic subcellular proteomes and their prokaryotic analogs’. **c** The basic AA contents of eukaryotic subcellular proteomes and their prokaryotic analogs’

To trace the changes of charged AA content in the macroevolution of prokaryotes to eukaryotes, we analyzed and compared them in 1051 prokaryotic genomes and 66 eukaryotic genomes (see detail in [17]). For acidic AA content, no significant difference was found between prokaryotes and eukaryotes genomes (*P* = 0.951) - the average acidic AA is 0.116 per AA for prokaryotes, and 0.115 per AA for eukaryotes; For basic AA, eukaryotes have a higher content than prokaryotes (*P* < 1E-3) - the average basic AA is 0.134 per AA for prokaryotes, and 0.143 per AA for eukaryotes (Fig. 3a and Additional file 1: Table S5).

### Evolutionary law of the charged AA contents between human subcellular proteomes and their prokaryotic analogs

In order to observe how various subcellular proteomes’ charged AA contents differ in the evolutionary process from prokaryotes to eukaryotes, we compared the charged AA contents between various human subcellular proteomes and their prokaryotic analogs. Because the human proteome is richest of subcellular annotations, and many cytoskeletal proteins were only identified in human until now. Ten human subcellular proteomes and their prokaryotic analogs were constructed between human and 158 mesophilic prokaryotes proteomes (Additional file 1: Table S4).

The charged AA contents of proteins were calculated and compared. For acidic AA, we observed that human transmembrane proteome’s acidic AA is higher than that in prokaryotes, and the mean content of human cytoskeleton and golgi proteomes were higher than their prokaryotic analogs (Fig. 3b and Additional file 1: Table S7). For basic AA, we observed that the average contents of almost all human subcellular proteomes were higher than their prokaryotic analogs (Fig. 3c and Additional file 1: Table S8).

### Charge characteristic of different cytoskeletal protein families

**Fig. 4:**
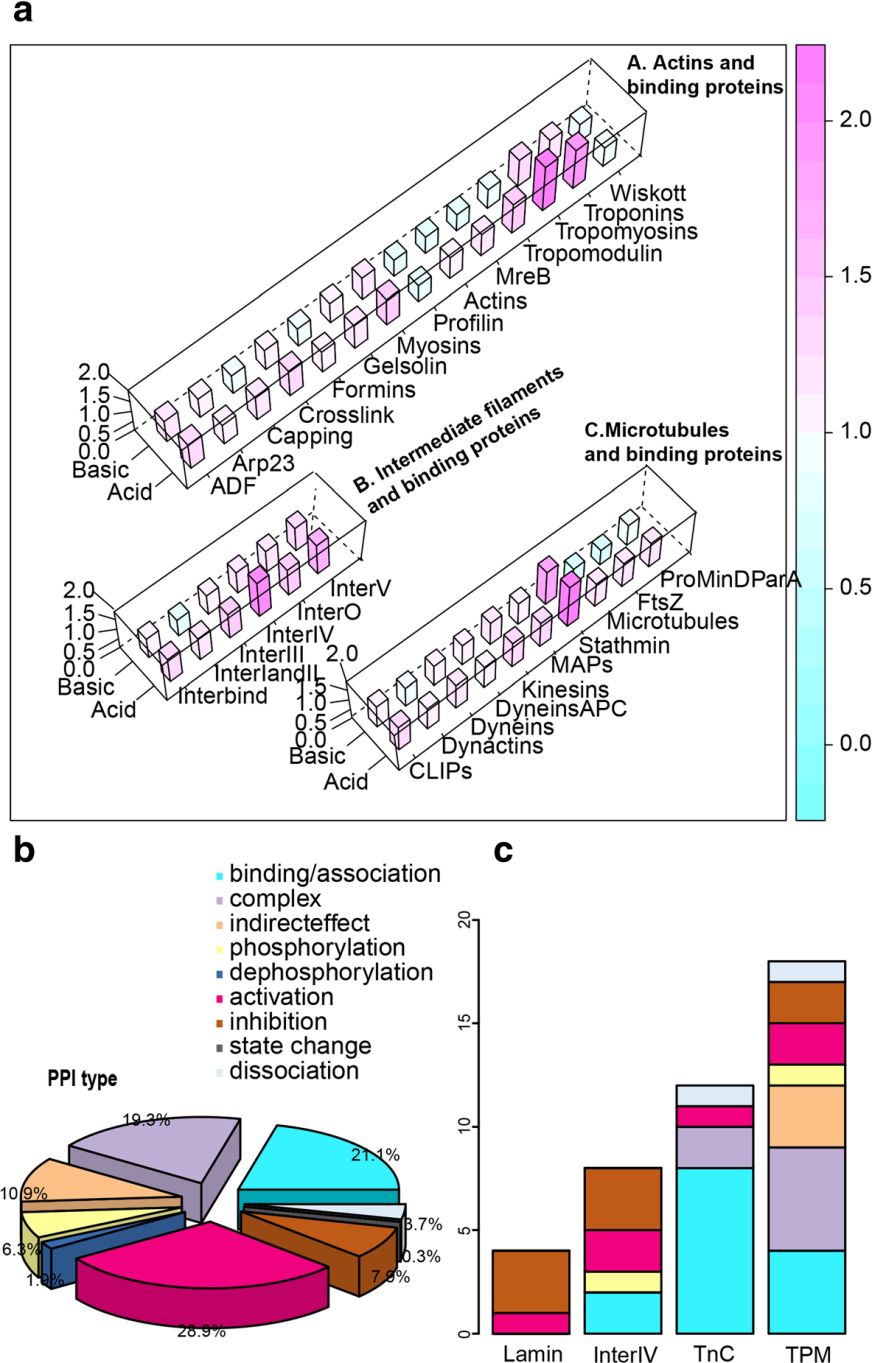
Charge characteristic and functional subdivision of the cytoskeletal protein families. **a** The charged amino acid contents of Actins, tubulins and intermediate filaments. To transform charged AA contents for each cytoskeletal protein, which were divided by the eukaryotes average acid (0.114955653) or basic AA (0.142665842) content. Effectively, this transformed the data and showed the difference between these proteins and eukaryotic proteins as a whole. Pink color (value > 1) shows the content higher than the mean content of the eukaryotes, blue color (value < 1) shows the content lower than the mean content of the eukaryotes. **b** Functional classification for all cytoskeletal proteins, by analysis their relationship with their associated upstream and downstream proteins. **c** Functional classification for Lamin, InterIV, TnC and TPM

In order to determine the charged AA contents of different cytoskeletal protein families, we retrieved cytoskeletal protein families from KEGG Orthology System (KO), which grouped different protein families based on homology. The cytoskeleton is made up of three kinds of protein filaments: actin filaments, intermediate filaments (IF) and microtubules, along with many other binding proteins. Charged AA contents of these families were then calculated and compared (Fig. 4a and Additional file 1: Tables S9). The results showed that 1) most of these families’ acidic AA and basic AA contents were higher (show in pink) than the average level of the overall eukaryotic proteins, and some were almost twice as high as the average level (e.g. the acidic AA contents of Troponins (TnC), Tropomyosins (TPM), Inter IV and Stathmin (Lamin); 2) Compared with basic content, acidic AA contents were much higher (more pink) than the average. Only Profilin and Wiskott’ acidic contents were slightly lower (light green); 3) For basic AA content, some cytoskeletal protein was slightly lower than average, including: A) the actin protein group, capping proteins, Formins, Profilin, Tropomodulin, Wiskott, actins and MreB; B) the intermediate filament protein group, only inter I and II; C) and the microtubule protein group, microtubulin, and four prokaryotic proteins: ftsZ, ParA, ParB, and MinD. 4)Almost all intermediate filaments (IF) group proteins have relatively higher charged AA contents than the average level of the overall eukaryotic proteins.

Interestingly, there is no significant difference in charged AA contents between MreB (prokaryotic actin) and actin proteins. And for ftsZ (prokaryotic microtubulin), which is nearly the same as that of microtubules (Fig. 4a and Additional file 1: Table S9).

### Functional subdivision of cytoskeletal proteins

In order to understand the functions of cytoskeletal proteins, we dissected their functions in the KEGG pathway [18]. We artificially analyzed 379 types of the relationships between each cytoskeletal protein and its upstream or downstream proteins one by one carefully (Additional file 1: Table S10). The results showed that the four largest categories were activation (28.9%); binding/association (21.1%); complex (19.3%); and indirect effect (10.9%), followed by other PPI types (7.9% inhibition, 6.3% phosphorylation, 3.7% dissociation, 1.9% dephosphorylation, and 0.3% state change) (Fig. 4b). An overview of each cytoskeleton’s functional relationships to its upstream and downstream was presented (Additional file 1: Table S10). InterV (Lamin), InterIV, Troponins (TnC) and TPM (Tropomyosins) were found as the top 4 highest acidic AA content proteins (Fig. 4b and Additional file 1: Table S9). Compared to Lamin and InterIV, TnC and TPM have been studied more because more PPI types were recorded (Additional file 1: Table S10). For them, we noticed that the two largest categories recorded were also precisely binding and (forming) complex (Fig. 4c).

### Electrostatic interactions of the cytoskeletal proteins

From the above analyses, we noticed that cytoskeletal proteins acidic AA content is the highest among all the investigated subcellular proteomes. In evolution, the average charged AA contents of human cytoskeletal proteins were higher than their prokaryotic analogs. Most of the cytoskeletal protein families possess higher charged AA contents than those of the average eukaryotic cell. Furthermore, the functions of cytoskeletal proteins were closely associated with binding, dissociation and building complexes. Here, we further explored the detailed functional mechanisms of the charged AAs, how they help cytoskeleton binding, dissociation and building complex.

### Electrostatic interaction at the primary structure level

**Fig. 5:**
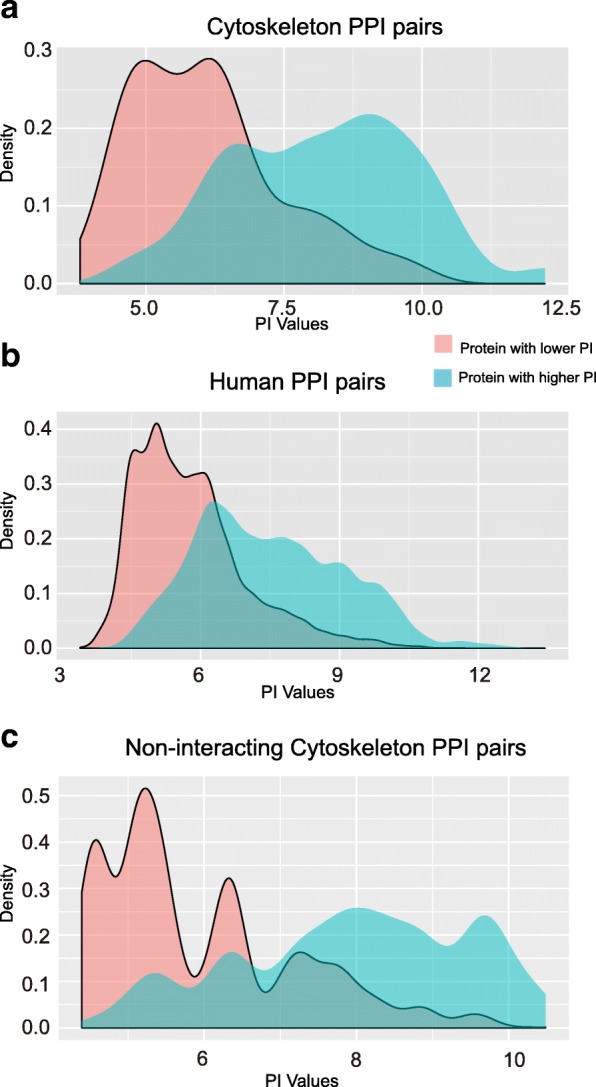
PIs (isoelectric points) distributions of protein-protein interaction (PPI) pairs. For each two interacting proteins’ PI value in a PPI pair, the lower PI value is showed in red and higher value in green. **a** PIs distributions of cytoskeleton PPI pairs. **b** PIs distributions of in vivo evidenced human PPI pairs. **c** PIs distributions of the PI difference distribution of non-interacting cytoskeletal protein pairs

First, we hypothesized that proteins in the cytoskeleton system interact with the other proteins by electrostatic interaction between net positive and negative charges of two protein sequences. To test this hypothesis, we calculated the theoretical PI (isoelectric point) between two proteins in each PPI (protein-protein interaction), then compared the two proteins PI distributions of human cytoskeleton PPI pairs (251) and all human PPI pairs evidenced in vivo (Fig. 5 and Additional file 1: Table S11). For the two proteins in each protein-protein interaction pair, there are two theoretical PI values. The lower PI value is shown in red and the higher one in green. For the cytoskeleton PPI pairs, it is interesting to note that two kinds of values showed a bimodal distribution, and there is an obvious segmentation near PI 7.0 (Fig. 5a). For the other human PPI pairs, the distribution of the two kinds PI overlapped a lot, and no obvious segmentation could be found near PI 7.0 (Fig. 5b). Next, to make a comparison of the PI difference distribution of cytoskeleton versus non-cytoskeleton PPI pairs, we use (PImax-PImin) to evaluate PI difference in one PPI pair. Results showed that PI difference was significantly larger in cytoskeleton PPI pairs than that in non-cytoskeleton PPI pairs (*P* value <3E-5) (Additional file 1: Table S12).

Furthermore, we showed the PI difference distribution between non-interacting cytoskeletal protein pairs (random sampling two proteins from non-interacting cytoskeletal proteins) (Fig. 5c). The lower PI value is shown in red and the higher one in green. For non-interacting cytoskeletal protein pairs, it is interesting to note that two kinds of values showed a multimodal distribution, not bimodal distribution. Two obvious peaks around 6.5 and 8 (Fig. 5c) suggested that many non-interacting cytoskeletal proteins are neutral. Another three peaks were around 4.5, 5.3, and 9.7, which is coincide with the results that the charged AA of cytoskeleton proteins were most abundant than the other subcellular located proteomes (Fig. 2c, d), and many cytoskeleton proteins’ charged amino acid content were much higher than average level (Fig. 4). Significant difference of PI difference (PImax-PImin) between cytoskeleton PPI and non-interacting cytoskeletal protein pairs was not supported by both of the tests (*P*>0.05 in Mann-Whitney Test, *P* = 0.004 in Kolmogorov-Smirnov test.) (Additional file 1: Table S12). In addition, since Mann-Whitney test is more robust [19], we choose to believe the result supported by Mann-Whitney Test when there is a discrepancy. Because the discrimination of interacting cytoskeletal proteins and non-interacting proteins were based on experimental data available now. This result indicated that more unknown interactions would be identified in future for those non-interacting cytoskeletal proteins.

### Electrostatic interaction at the 3D structure level

We then hypothesized that proteins in the cytoskeleton system might also interact with the other proteins through electrostatic interaction on protein-protein interaction interfaces. To test this hypothesis, we calculated the charged AA of interfaces among protein 3D structures. Cytoskeletal proteins with 3D structures in PDB were retrieved, and charged AA contents of PPI interfaces in the 3D structures were then calculated and compared with that of random non-cytoskeleton protein-protein interaction interfaces. Our results showed that a significantly higher content of Glu (E) in the cytoskeleton PPI interfaces (Table 1). To demonstrate that higher content of Glu (E) in the cytoskeleton PPI interfaces is not just due to the generally higher level of charged amino acid in cytoskeletal proteins, we further compared charged AA contents of non-PPI parts and PPI of cytoskeleton complex sequences. The results showed that the average Glu content of PPI interfaces of cytoskeleton were higher than that of the non-PPI interfaces of cytoskeleton proteins (Additional file 1: Table S13).

**Table 1:** The charged amino acid (AA) content of cytoskeleton PPIs and random PPIs from PDB database

AA Type	Mean content of Cytoskeleton PPI	Mean content of Random PPI	*P* value	
			Mann-Whitney Test, I-tailed	Kolmogorov-Smirnov Test, l-tailed
Acidic AA	0.169	0.141	0.118839	0.138833
Glu	0.115	0.078	0.043527	0.004701
Asp	0.054	0.063	0.257816	0.482385
Basic AA	0.188	0.171	0.280319	0.457611
Lys	0.080	0.062	0.088308	0.199456
Arg	0.086	0.078	0.383006	0.175169
His	0.021	0.031	0.192222	0.485181

## Discussion

Studies of stoichiogenomics have primarily focused on the elements usage in proteins and nucleic acids [10, 20]. Like element, monomers and functional groups of monomers are also material composition of proteins or nucleic acids. Here we updated the definition of stoichiogenomics, which aims to research patterns of elements, functional groups, monomers and measurable chemical properties of proteins, nucleic acids in genomics. Then we applied stoichiogenomics to subcellular research to examine differential patterns of elements and charged AA compositions of diverse subcellular proteomes.

The extracellular proteins are commonly synthesized in cytoplasm, modified in the endoplasmic reticulum and Golgi, and secreted by a cell [21–23]. Compared to other subcellular proteomes investigated, we found the hydrogen content of the extracellular proteome is the lowest, and that its sulfur content is high. High sulfur content is due to the abundant disulfide bridges in the extracellular proteins [24]. However, we could not explain the lowest hydrogen content of the extracellular proteins in this article. In addition, in correlation analysis of results obtained from single and dual location proteins, the low correlation of sulfur contents should be caused by the extracellular proteins, which are synthesized in cytoplasm and modified in the endoplasmic reticulum and Golgi. Therefore, in the location annotation, the extracellular proteins were duplicated in many subcellular locations. The extracellular proteins’ own high sulfur content might bias the sulfur results obtained from dual location proteins.

In our study, we found that the nuclear proteins have the highest basic AA contents among all the investigated subcellular proteomes. The highest basic AA contents of the nuclear proteins might help their binding with negatively charged DNA existing in the nucleus. For example, the nucleus has an abundance of histones, which are highly alkaline proteins [22, 23]. In addition, we found that the high nitrogen content of the nuclear proteomes is highly correlated with their high basic AA content, since nitrogen only appears in amide and basic AAs.

The cytoskeleton has the highest acidic AA content among all the investigated subcellular proteomes. For basic AA content, the cytoskeleton proteome is the second highest. Results obtained from single and dual location proteins are consistent. These suggest that acidic AA content could be used as a good predictor for cytoskeletal proteins, and basic AA content for the nucleus. The charged AA contents of other subcellular locations are diverse and often intersected. In addition, results obtained from model organisms are more regular than non-model organisms. This bias might be caused by uneven data distribution, because subcellular location annotation is very rare in non-model organisms. A universal trend could be obtained when more subcellular annotation data are available.

Comparing to prokaryotic proteins, eukaryotic proteins basic AA content is significantly increased. It means for the entire, eukaryotic proteins are tending to be more basic than acidic. For the cytoskeleton, we found the average contents of both acidic and basic AA content of human cytoskeletal proteins were higher than their prokaryotic analogs. Under the background of increased basic characteristic of entire eukaryotic proteins, more acidic AA in cytoskeletal proteins made them more distinguished in eukaryotic cell.

Most of the cytoskeletal protein families possess higher acidic and basic AA contents than that of eukaryotic average level. Especially for the acidic contents of Troponins, Tropomyosins, Inter IV and Stathmin, which were two times higher than the average level. These results could be useful for diagnosis and therapy for various cytoskeleton related diseases. In addition, we observed that the average contents of charged AA of cytoskeleton were higher than their prokaryotic analogs in evolutionary law analysis. Here, subdivision of protein families suggested that the increase is mainly due to the newly originated cytoskeletal protein in eukaryotes, including IF protein [21, 24], Troponins [23], Tropomyosins [22] and Stathmin [21], whose acidic AA contents were much higher. However, the charged AA content of prokaryotic originated protein, like actin and microtubulin, were almost as same as their ancients’.

Functionally subdivision results suggested that cytoskeletal proteins were closely associated with activation, binding, dissociation and building complex categories. Activation is a much bigger concept, including phosphorylation, acetylation, methylation, ubiquitination, electrostatic interactions on active surface and so on [25]. As the major functional category of the cytoskeleton, the binding, dissociation and building complex functions are comparatively more specific concepts, and compatible with cytoskeleton’s structure and location characteristic. Because cytoskeleton need continuous binding, dissociation, and building complex to supports cell and provides shape, enables it to move, and serves purposes in intracellular transport, polarity and cellular division [26, 27]. This hypothesis is evidenced in TnC and TPM, whose functions were focused on binding and building complex. Less data of Lamin and InterIV means they are still poor studied. We predict that more binding and building complex relationships would be identified between them and their upstreams and downstreams in future.

Theoretical PI distribution of cytoskeletal protein pairs showed an obvious match of negative and positive charge between two interacting primary sequences. Electrostatic interaction analysis in protein 3D structures showed that the cytoskeleton complexes use more Glu (E) among PPI interfaces than that of the other complexes. It is known that the amino acid composition of PPI interfaces are frequently hydrophobic and electrostatic complementary [28], but no amino acid usage bias exists in different PPI interfaces according to previous research [29]. However, in this study we found more Glu (E) in PPI interfaces of cytoskeleton complexes than those of other random selected complexes. It further enhanced the effect of electrostatic forces in cytoskeletal protein. A combined mechanism of increased electrostatic interaction between both primary sequences and PPI interfaces showed an efficient and flexible use of charged AA in cytoskeletal proteins.

The cytoskeleton is an open bones system, not like other organelles that are relatively closed and surrounded by two membranes (nucleus, mitochondria, and chloroplast) or one membrane (endoplasmic reticulum, Golgi). Proteins in organelles surrounded by membranes have natural physical barriers, while cytoskeletal proteins have no physical barriers in cell. They are widely distributed in the cell to keep the stability of the cell physiologically and structurally [6]. At first, as cytoskeleton performs multiple functions and widely distributed, it interacts with various molecules within a cell’s cytoplasm. Rich acidic AA benefits these interactions definitely. Secondly, since eukaryotic proteins are tend to be more basic, acidic AA is necessary for the individual cytoskeletal protein molecules to carry the same electric charge and help prevent self-aggregation [30, 31]; Thirdly, it is necessary for the individual cytoskeletal protein molecules keeping net charge and helping specifically recognition [32] among cytoskeletal proteins. Previous biochemistry research has shown that microfilament and microtubule moves in a treadmilling model [8, 33]. By stoichiogenomic analysis, our study revealed charged characteristics and its evolutionary law of the cytoskeleton, and provided functional interpretation of how charged AAs played role in whole cytoskeleton system at both sequences and structures levels.

## Conclusions

In this study, we renewed the definition of stoichiogenomics to research different patterns of elements, functional groups, monomer, and measurable chemical properties of proteins, nucleic acids. Then we applied it to examine differential patterns of element and charged AA compositions of proteins in different subcellular locations. This is the first time that systematic insights have been gained into stoichiogenomic characteristics of subcellular proteome and evolutionary law, electrostatic interaction of how charged AA played role in whole cytoskeleton system at both primary and higher structures levels. These studies provide a subcellular stoichiogenomics blueprint and functional interpretation of the binding/association and complexing of the cytoskeleton. It helps us to better understand protein’s evolutionary adaptation to various subcellular conditions in terms of element and charged AA compositions, and functional interpretation of macromolecular machinery. The results obtained here could be used for subcellular locations prediction, protein function prediction, and contributed to basic cell biology of the cytoskeleton. Understanding the basic cell biology of the cytoskeleton would further contributed to the investigation, diagnosis, and therapy for various cytoskeleton related disease. In addition, this study lays a foundation for applying stoichiogenomics research in more species and fields, such as the stoichiogenomic characteristics of cancer microenvironment and further used in cancer prediction, and some unresolved evolutionary and species classification problems [10, 11].

## Methods

### Retrieve sequence and sort them into different subcellular locations

The general approach for sorting protein sequences according to their subcellular location annotations were as followings (Additional file 1: Figure S1) [1]: all available eukaryotic proteins (400,854) were downloaded from Swiss-prot (UniProt Release 14.4) [34] [2]. Only eukaryotic proteins with subcellular locations evidenced by experiments (46,231) were chosen for further analyses [3]. Identifying single location and dual location proteins: sequences with only one subcellular location annotation were regarded as single subcellular located proteins (Additional file 1: Table S1); Sequences with more than one subcellular location annotation were regarded as dual subcellular located proteins (Additional file 1: Table S2). Considering the repeat named of cytoskeletal proteins, “cytoskeleton, nucleus” and “cytoskeleton, cytoplasm” in annotation were recorded as “cytoskeleton” in both cases [4]. Protein sequences were then sorted according to their species resources (Additional file 1: Table S3), including: *Saccharomyces cerevisiae (*Baker’s yeast*), Schizosaccharomyces pombe (*Fission yeast*), Arabidopsis thaliana, Bos taurus* (Bovine)*, Gallus gallus* (Chicken), *Caenorhabditis elegans* (Elegans), *Drosophila melanogaster* (Fly)*, Xenopus tropicalis* (Frog)*, Sus scrofa* (Pig)*, Rattus norvegicus* (Rat)*, Mus musculus* (Mouse)*,* and *Homo sapiens* (Human), to ensure that the amount of sequences in each species were larger than 300 [5]. Protein sequences in each species were then sorted into different subcellular location categories. According to Swiss-prot annotation, there are 17 kinds of subcellular location categories. To ensure that the final data size of different location categories in each species is at least 1 (sequence), the following location categories were finally kept: cytoplasm, cytoskeleton, endoplasmic reticulum, extracellular, golgi, membrane, mitochondria, nucleus, and transmembrane.

### Construct homolog protein sets between human and prokaryotic genomes

We retrieved 158 mesophilic prokaryotic genomes from GenBank (ftp://ftp.ncbi.nih.gov/genomes) (Additional file 1: Table S4). Mesophiles are organisms with growth temperature among 15 °C–50 °C according to PGTdb [35]. Since previous studies have showed that charged AA and oxygen contents have a close correlation with thermophilic prokaryotes [17, 36]. Hyperthermophiles, thermophiles and psychrophiles were not included to eliminate biases.

In order to more accurately identify prokaryotic ancestor sequences of proteins in different subcellular location categories, Bi-directional BLAST were conducted between human proteins with different categories and 158 mesophilic prokaryotic genomes by the method we used before [37]. Bi-directional best hit has been widely used to identify the orthologous genes between different genomes [38].

At first, human proteins were used as queries to compare with each prokaryotic translated genome by BLASTP (E-value < = 1e-5), and sequences producing significant alignments were calculated and kept for further analysis. Secondly, we performed a reciprocal search with BLASTP (E-value < = 1e-5) using each prokaryotic translated genomes as the queries, respectively. Pairs of sequences that were each other’s best hit were identified in both directions and regarded as putative 1:1 ortholog genes between human and prokaryotic genomes.

### Classify cytoskeletal proteins into different families

Orthology (KO) System in the Kyoto Encyclopedia of Genes and Genomes (KEGG) [18] were used to retrieve different cytoskeletal proteins families (Additional file 1: Tables S9).

### Retrieve protein-protein interaction (PPI) pairs

Human protein interaction data (38, 788 pairs) were retrieved from HPRD (Human Protein Reference Database, release 9) [39]. Among them, 18, 584 PPI pairs were evidenced in vivo.

### Retrieve protein-protein interaction (PPI) segments from 3D structure

Since subcellular location information in PDB (The Protein Data Bank) [40] is not enough, we used cytoskeleton system proteins evidenced by experiments (in Swiss-prot) as queries to search sequences in PDB by Blastp with stringent cutoff (E-value < = 1e-10). The hit sequences were considered as cytoskeletal proteins (or complexes) in PDB.

The protein-protein interaction interfaces in 3D structure were first retrieved from PDBsum [41]. These interfaces contain both hydrogen bonds residues and the non-hydrogen bonds residues. Then, these interfaces were divided into cytoskeleton protein-protein interaction interfaces and non-cytoskeleton PPI interfaces. At last, 132 cytoskeleton protein-protein interaction interfaces from 37 cytoskeleton complexes were used to compare with non-cytoskeleton PPI interfaces selected from 2000 random complexes.

### Calculation of element content

Average content of the five kinds of elements (carbon, hydrogen, oxygen, nitrogen and sulfur) in a full protein sequence was estimated as follows: [∗*content*] =  ∑ *wi* × *pi*/*L*. The wi is the number of * element (or functional group) on the AA side chain (ranging from 0 to 10), pi is the content of the i-th AA, and L is the sequence length. The content of an element is the mean of all elements in all sequences in one proteome or one orthologous group. All data were calculated by using published method [7, 17].

### Amino acid content and PI calculation

Average contents of charged AAs in a full protein sequence were estimated as follows:

- One sequence’s [acidic AA content] = [sum of acidic AA]/L.
- One sequence’s [basic AA content] = [sum of basic AA]/L.

Where acidic AA includes: Aspartic acid (Asp, D), Glutamic acid (Glu, E); basic AA includes: lysine (Lys, K), arginine (Arg, R) and histidine (His, H); L is the sequence length. The content of acidic (or basic) AA is the mean of the acidic (or basic) AA in all sequences, for each subcellular location in each species. All data were calculated by using published method [17, 36].

For PI (isoelectric point) calculation, we used PI computing tool on the expasy (http://web.expasy.org/compute_pi/). To evaluate PI difference in one PPI pair, we use PImax-PImin. PImax means the larger PI value of two proteins in one PPI pair. PImin means the smaller PI value of two proteins in one PPI pair.

### Statistical tests and visualizations

Comparisons of two independent samples were conducted by Mann-Whitney and Kolmogorov-Smirnov tests in SPSS 19.0 statistics software (SPSS Inc., Chicago, IL). Multiple analyses were conducted by Turkey’s multiple comparisons in R statistical environment (v3.3.1) [42]. For robustness and consistency, we only considered in this work significant differences at the probability level of *p* < 0.05 by two statistically methods. The visualizations were prepared by using basic drawing functions, ggplot2 (2.2.1) [43], circlize (0.4.3), lattice (0.20–35), latticeExtra (0.6–28) in R statistical environment (v3.3.1) [42].

## Additional file

Additional file 1: **Tables S1** to **S9**, **S11** to **S13** and **Figures S1** to **S10**. **Table S10**: The protein-protein interaction (PPI) types between each cytoskeleton protein and its associated upstream and downstream proteins in KEGG pathway. (ZIP 2429 kb)

## Abbreviations

AA: Amino acid
PI: Isoelectric point
PPIs: Protein-protein interactions
