# Supplementary material for: Subcellular stoichiogenomics reveal cell evolution and electrostatic interaction mechanisms in cytoskeleton
Source: BMC Genomics. 2018 Jun 18;19:469. doi: 10.1186/s12864-018-4845-0 (PMC6006717; doi:10.1186/s12864-018-4845-0)

Additional files for

**Subcellular stoichiogenomics reveal cell evolution and electrostatic interaction mechanisms in cytoskeleton**

Yu-Juan Zhang^1,2^, Chengxu Zhu^1^, Yiran Ding^1^，Zheng-Wen Yan^1^, Gong-Hua Li^2^, ,Yang Lan^1^, Jian-Fan Wen^2*^, Bin Chen^1*^

*^1^ Institute of Entomology and Molecular Biology, College of Life Sciences, Chongqing Normal University, Shapingba, Chongqing 401331, PR China*

*^2^ State Key Laboratory of Genetic Resources and Evolution, Kunming Institute of Zoology, Chinese Academy of Sciences, Kunming, Yunnan Province 650223, PR China.*

*Corresponding author. E-mail: [c_bin@hotmail.com](mailto:c_bin@hotmail.com)

[wenjf@mail.kiz.ac.cn](mailto:wenjf@mail.kiz.ac.cn)

**This PDF file includes**

Tables. S1 to S9, S11 to S13.

Figures. S1 to S10.

**Other Supplementary Material for this manuscript includes the following:**

**Table S10**: The protein-protein interaction (PPI) types between each cytoskeleton protein and its associated upstream and downstream proteins in KEGG pathway.

Additional files

**Tables**

**Tables S1: Statistics of single located eukaryotic proteins with different subcellular location annotations used in this analysis. All annotations were evidenced by experiments retrieved from Swiss-prot.**

| **Organism** | **Cytoplasm** | **Cytoskeleton** | **ER** | **Golgi** | **Membrane** | **Mitochondrion** | **Nucleus** | **Secretory** | **Transmembrane** |
| --- | --- | --- | --- | --- | --- | --- | --- | --- | --- |
| *Saccharomyces cerevisiae* | 503 | 26 | 151 | 63 | 423 | 457 | 717 | 7 | 377 |
| *Schizosaccharomyces pombe* | 295 | 25 | 111 | 27 | 106 | 181 | 445 | 3 | 84 |
| *Arabidopsis thaliana* | 110 | 19 | 33 | 22 | 308 | 128 | 269 | 18 | 221 |
| *Bos taurus* | 83 | 14 | 8 | 7 | 244 | 183 | 60 | 301 | 155 |
| *Gallus gallus* | 59 | 13 | 6 | 2 | 137 | 27 | 153 | 129 | 72 |
| *Caenorhabditis elegans* | 69 | 7 | 11 | 5 | 55 | 17 | 106 | 43 | 36 |
| Drosophila*melanogaster* | 101 | 17 | 15 | 18 | 114 | 39 | 309 | 420 | 63 |
| *Xenopus tropicalis* | 52 | 11 | 6 | 1 | 90 | 9 | 205 | 91 | 53 |
| *Sus scrofa* | 65 | 5 | 4 | 2 | 110 | 39 | 31 | 217 | 74 |
| *Rattus norvegicus* | 261 | 27 | 51 | 29 | 803 | 159 | 263 | 368 | 559 |
| *Mouse* | 389 | 38 | 93 | 50 | 1234 | 191 | 783 | 583 | 768 |
| *Human* | 692 | 66 | 173 | 104 | 1924 | 386 | 1395 | 735 | 1267 |

**Tables S2: Statistics of dual located eukaryotic proteins with different subcellular location annotations used in this analysis. All annotations were evidenced by experiments retrieved from Swiss-prot.**

| **Organism** | **Cytoplasm** | **Cytoskeleton** | **ER** | **Golgi** | **Membrane** | **Mitochondrion** | **Nucleus** | **Secretory** | **Transmembrane** |
| --- | --- | --- | --- | --- | --- | --- | --- | --- | --- |
| *Saccharomyces cerevisiae* | 1171 | 50 | 228 | 129 | 1085 | 558 | 1296 | 44 | 708 |
| *Schizosaccharomyces pombe* | 925 | 37 | 149 | 72 | 351 | 209 | 1103 | 9 | 279 |
| *Arabidopsis thaliana* | 231 | 31 | 38 | 34 | 540 | 160 | 345 | 91 | 335 |
| *Bos taurus* | 155 | 21 | 23 | 13 | 428 | 210 | 72 | 313 | 312 |
| *Gallus gallus* | 116 | 26 | 20 | 9 | 226 | 31 | 174 | 136 | 174 |
| *Caenorhabditis elegans* | 146 | 29 | 15 | 12 | 121 | 21 | 151 | 44 | 86 |
| *Drosophila melanogaster* | 231 | 39 | 22 | 25 | 223 | 48 | 386 | 125 | 150 |
| *Xenopus tropicalis* | 116 | 19 | 9 | 3 | 140 | 10 | 252 | 93 | 120 |
| *Sus scrofa* | 82 | 8 | 18 | 7 | 158 | 48 | 40 | 228 | 125 |
| *Rattus norvegicus* | 563 | 53 | 159 | 77 | 1297 | 198 | 389 | 403 | 991 |
| *Mouse* | 1012 | 150 | 258 | 174 | 490 | 325 | 1327 | 600 | 1516 |
| *Human* | 1907 | 282 | 339 | 248 | 3162 | 476 | 2196 | 833 | 2457 |

**Tables S3: 12 eukaryotic species investigated in the analysis. Protein: total number of predicted proteins; Mean sequence length: Mean length of all proteins in an organism; Organism: species identification.**

| **Protein** | **Mean sequence length** | **Organism** |
| --- | --- | --- |
| 32615 | 566 | *Arabidopsis thaliana* |
| 33684 | 528 | *Bos taurus* |
| 22844 | 577 | *Caenorhabditis elegans* |
| 20492 | 479 | *Drosophila melanogaster* |
| 18532 | 484 | *Gallus gallus* |
| 11628 | 491 | *Gibberella zeae* |
| 22997 | 409 | *Homo sapiens* |
| 34966 | 471 | *Mus musculus* |
| 29570 | 622 | *Rattus norvegicus* |
| 5882 | 618 | *Saccharomyces cerevisiae* |
| 5020 | 765 | *Schizosaccharomyces pombe* |
| 20180 | 469 | *Sus scrofa* |

**Tables S4: 158 mesophilic prokaryote species investigated in the analysis. Taxonomy: the taxonomic status of the species; Protein: total number of predicted proteins; Organism: species identification; Lifestyle: organism’s lifestyle, whenever available (PGTdb).**

| **Taxonomy** | **Protein** | **Organism** |
| --- | --- | --- |
| Actinobacteria | 1261 | *Gardnerella_vaginalis_409_05_uid43211* |
| Actinobacteria | 2551 | *Corynebacterium_aurimucosum_ATCC_700975_uid59409* |
| Actinobacteria | 1806 | *Bifidobacterium_longum_BBMN68_uid60163* |
| Actinobacteria | 1731 | *Arcanobacterium_haemolyticum_DSM_20595_uid49489* |
| Actinobacteria | 8197 | *Rhodococcus_opacus_B4_uid13791* |
| Actinobacteria | 2375 | *Propionibacterium_freudenreichii_shermanii_CIRM_BIA1_uid49535* |
| Actinobacteria | 4909 | *Nocardioides_JS614_uid58149* |
| Actinobacteria | 8913 | *Catenulispora_acidiphila_DSM_44928_uid59077* |
| Actinobacteria | 4523 | *Arthrobacter_FB24_uid58141* |
| Actinobacteria | 7197 | *Saccharopolyspora_erythraea_NRRL_2338* |
| Actinobacteria | 7676 | *Streptomyces_avermitilis_MA_4680_uid57739* |
| Actinobacteria | 5120 | *Mycobacterium_avium_104_uid57693* |
| Actinobacteria | 2236 | *Micrococcus_luteus_NCTC_2665_uid59033* |
| Actinobacteria | 5935 | *Nocardia_farcinica_IFM_10152_uid58203* |
| Actinobacteria | 3678 | *Cellulomonas_flavigena_DSM_20109_uid48821* |
| Actinobacteria | 6222 | *Micromonospora_aurantiaca_ATCC_27029_uid42501* |
| Actinobacteria | 1261 | *Gardnerella_vaginalis_409_05_uid43211* |
| Actinobacteria | 2551 | *Corynebacterium_aurimucosum_ATCC_700975_uid59409* |
| Alphaproteobacteria | 1374 | *Rickettsia_conorii_Malish_7_uid57633* |
| Alphaproteobacteria | 1736 | *Zymomonas_mobilis_ZM4_uid58095* |
| Alphaproteobacteria | 3011 | *Erythrobacter_litoralis_HTCC2594_uid58299* |
| Alphaproteobacteria | 4799 | *Ochrobactrum_anthropi_ATCC_49188_uid58921* |
| Alphaproteobacteria | 4129 | *Roseobacter_denitrificans_OCh_114_uid58597* |
| Alphaproteobacteria | 3512 | *Hyphomicrobium_denitrificans_ATCC_51888_uid50325* |
| Alphaproteobacteria | 5963 | *Rhizobium_etli_CFN_42_uid58377* |
| Alphaproteobacteria | 2664 | *Gluconobacter_oxydans_621H_uid58239* |
| Alphaproteobacteria | 6213 | *Sinorhizobium_medicae_WSM419_uid58549* |
| Alphaproteobacteria | 3565 | *Rhodomicrobium_vannielii_ATCC_17100_uid43247* |
| Alphaproteobacteria | 3122 | *Nitrobacter_winogradskyi_Nb_255_uid58295* |
| Alphaproteobacteria | 3034 | *Brucella_melitensis_biovar_Abortus* |
| Alphaproteobacteria | 7272 | *Mesorhizobium_loti_MAFF303099_uid57601* |
| Alphaproteobacteria | 3937 | *Novosphingobium_aromaticivorans_DSM_12444_uid57747* |
| Alphaproteobacteria | 4878 | *Rhodopseudomonas_palustris_BisA53_uid58445* |
| Alphaproteobacteria | 3722 | *Oligotropha_carboxidovorans_OM5_uid59155* |
| Alphaproteobacteria | 3327 | *Brevundimonas_subvibrioides_ATCC_15264_uid42117* |
| Alphaproteobacteria | 4559 | *Magnetospirillum_magneticum_AMB_1_uid58527* |
| Alphaproteobacteria | 5345 | *Sphingomonas_wittichii_RW1_uid58691* |
| Alphaproteobacteria | 4394 | *Sphingobium_japonicum_UT26S_uid47077* |
| Alphaproteobacteria | 6309 | *Azospirillum_B510_uid46085* |
| Alphaproteobacteria | 5077 | *Paracoccus_denitrificans_PD1222_uid58187* |
| Alphaproteobacteria | 3737 | *Caulobacter_crescentus_CB15_uid57891* |
| Alphaproteobacteria | 5035 | *Xanthobacter_autotrophicus_Py2_uid58453* |
| Alphaproteobacteria | 3854 | *Phenylobacterium_zucineum_HLK1_uid58959* |
| Alphaproteobacteria | 4003 | *Rhodospirillum_centenum_SW_uid58805* |
| Alphaproteobacteria | 3642 | *Rhodobacter_capsulatus_SB_1003_uid47509* |
| Alphaproteobacteria | 3852 | *Gluconacetobacter_diazotrophicus_PAl_5_FAPERJ* |
| Alphaproteobacteria | 6692 | *Methylobacterium_4_46_uid58843* |
| Aminobacterium | 1876 | *Aminobacterium_colombiense_DSM_1261_uid47083* |
| Betaproteobacteria | 2461 | *Nitrosomonas_europaea_ATCC_19718_uid57647* |
| Betaproteobacteria | 2805 | *Nitrosospira_multiformis_ATCC_25196_uid58361* |
| Betaproteobacteria | 2002 | *Neisseria_gonorrhoeae_FA_1090_uid57611* |
| Betaproteobacteria | 2753 | *Methylobacillus_flagellatus_KT_uid58049* |
| Betaproteobacteria | 4891 | *Comamonas_testosteroni_CNB_1_uid29203* |
| Betaproteobacteria | 4407 | *Chromobacterium_violaceum_ATCC_12472_uid58001* |
| Betaproteobacteria | 2867 | *Thiobacillus_denitrificans_ATCC_25259_uid58189* |
| Betaproteobacteria | 6040 | *Delftia_acidovorans_SPH_1_uid58703* |
| Betaproteobacteria | 3226 | *Ralstonia_solanacearum_CFBP2957_uid50545* |
| Betaproteobacteria | 3978 | *Thauera_MZ1T_uid58987* |
| Betaproteobacteria | 3989 | *Azoarcus_BH72* |
| Betaproteobacteria | 5027 | *Bordetella_petrii* |
| Betaproteobacteria | 6279 | *Variovorax_paradoxus_10_uid59437* |
| Betaproteobacteria | 4202 | *Burkholderia_pseudomallei_MSHR346* |
| CFB group bacteria | 5017 | *Flavobacterium_johnsoniae_UW101_uid58493* |
| CFB group bacteria | 4816 | *Bacteroides_thetaiotaomic on_VPI-5482* |
| CFB group bacteria | 2296 | *Prevotella_melaninogenica_ATCC_25845_uid51377* |
| CFB group bacteria | 3085 | *Fibrobacter_succinogenes_S85* |
| CFB group bacteria | 4252 | *Pedobacter_heparinus_DSM_2366_uid59111* |
| CFB group bacteria | 6938 | *Spirosoma_linguale_DSM_74_uid43413* |
| CFB group bacteria | 2327 | *Prosthecochloris_aestuarii_DSM_271_uid58151* |
| CFB group bacteria | 2043 | *Chlorobaculum_parvum_NCIB_8327_uid59185* |
| CFB group bacteria | 2434 | *Chlorobium_limicola_DSM_245_uid58127* |
| Chlamydias | 1119 | *Chlamydophila_pneumoniae_AR39_uid57809* |
| Chlamydias | 911 | *Chlamydia_muridarum_Nigg_uid57785* |
| Chloroflexi | 5279 | *Herpetosiphon_aurantiacus_ATCC_23779_uid58599* |
| Cyanobacteria | 6312 | *Microcystis_aeruginosa_NIES_843_uid59101* |
| Deinococcus-Thermus | 3167 | *Deinococcus_radiodurans_R1_uid57665* |
| Delta/epsilon proteobacteria | 1545 | *Campylobacter_lari_RM2100_uid58115* |
| Delta/epsilon proteobacteria | 1573 | *Helicobacter_pylori_26695_uid57787* |
| Delta/epsilon proteobacteria | 2043 | *Wolinella_succinogenes* |
| Delta/epsilon proteobacteria | 3436 | *Desulfomicrobium_baculatum_DSM_4028_uid59217* |
| Delta/epsilon proteobacteria | 2620 | *Desulfurivibrio_alkaliphilus_AHT2_uid49487* |
| Delta/epsilon proteobacteria | 7316 | *Myxococcus_xanthus_DK_1622_uid58003* |
| Delta/epsilon proteobacteria | 2356 | *Desulfovibrio_desulfuricans_ATCC_27774_uid59213* |
| Archaea | 4212 | *Natrialba_magadii_ATCC_43099_uid46245* |
| Archaea | 5113 | *Haloterrigena_turkmenica_DSM_5511_uid43501* |
| Archaea | 2646 | *Haloquadratum_walsbyi_DSM_16790_uid58673* |
| Archaea | 4243 | *Haloarcula_marismortui_ATCC_43049_uid57719* |
| Archaea | 3560 | *Halorubrum_lacusprofundi_ATCC_49239_uid58807* |
| Archaea | 2820 | *Natronomonas_pharaonis_DSM_2160_uid58435* |
| Archaea | 1535 | *Methanosphaera_stadtmanae_DSM_3091_uid58407* |
| Archaea | 4015 | *Haloferax_volcanii_DS2_uid46845* |
| Archaea | 1793 | *Methanobrevibacter_smithii_ATCC_35061_uid58827* |
| Archaea | 1987 | *Methanohalophilus_mahii_DSM_5219_uid47313* |
| Archaea | 2273 | *Methanococcoides_burtonii_DSM_6242_uid58023* |
| Archaea | 1678 | *Methanococcus_vannielii_SB_uid58767* |
| Archaea | 3139 | *Methanospirillum_hungatei_JF_1_uid58181* |
| Archaea | 1741 | *Methanocorpusculum_labreanum_Z_uid58785* |
| Archaea | 2490 | *Methanoculleus_marisnigri_JR1_uid58561* |
| Archaea | 3624 | *Methanosarcina_barkeri_Fusaro_uid57715* |
| Archaea | 2785 | *Methanoplanus_petrolearius_DSM_11571_uid52695* |
| Firmicutes | 1813 | *Finegoldia_magna_ATCC_29328_uid58867* |
| Firmicutes | 3811 | *Butyrivibrio_proteoclasticus_B316_uid51489* |
| Firmicutes | 2765 | *Eubacterium_eligens_ATCC_27750_uid59171* |
| Firmicutes | 2615 | *Staphylococcus_aureus_COL_uid57797* |
| Firmicutes | 3043 | *Listeria_innocua* |
| Firmicutes | 3847 | *Clostridium_acetobutylicum_ATCC_824_uid57677* |
| Firmicutes | 3264 | *Enterococcus_faecalis_V583_uid57669* |
| Firmicutes | 2270 | *Streptococcus_sanguinis_SK36_uid58381* |
| Firmicutes | 2473 | *Lactococcus_lactis_KF147* |
| Firmicutes | 1755 | *Pediococcus_pentosaceus_ATCC_25745_uid57981* |
| Firmicutes | 2005 | *Leuconostoc_mesenteroides_ATCC_8293_uid57919* |
| Firmicutes | 1610 | *Lactobacillus_helveticus_DPC_4571_uid58761* |
| Firmicutes | 3922 | *Bacillus_amyloliquefaciens_DSM7_uid53535* |
| Firmicutes | 1844 | *Veillonella_parvula_DSM_2008* |
| Firmicutes | 1691 | *Oenococcus_oeni_PSU_1_uid59417* |
| Firmicutes | 6213 | *Paenibacillus_JDR_2_uid59021* |
| Firmicutes | 4883 | *Desulfitobacterium_hafniense_DCB_2_uid57749* |
| Gammaproteobacteria | 4281 | *Pseudoalteromonas_atlantica_T6c_uid58283* |
| Gammaproteobacteria | 5489 | *Photobacterium_profundum_SS9* |
| Gammaproteobacteria | 1792 | *Haemophilus_influenzae_86_028NP_uid58093* |
| Gammaproteobacteria | 3662 | *Proteus_mirabilis* |
| Gammaproteobacteria | 3190 | *Legionella_pneumophila_2300_99_Alcoy_uid48801* |
| Gammaproteobacteria | 3712 | *Acinetobacter_baumannii_AYE* |
| Gammaproteobacteria | 4272 | *Marinobacter_aquaeolei_VT8_uid59419* |
| Gammaproteobacteria | 4474 | *Xenorhabdus_nematophila_ATCC_19061_uid49133* |
| Gammaproteobacteria | 2015 | *Pasteurella_multocida_Pm70_uid57627* |
| Gammaproteobacteria | 3693 | *Vibrio_cholerae_M66_2_uid59355* |
| Gammaproteobacteria | 2369 | *Mannheimia_succiniciproducens_MBEL55E_uid58197* |
| Gammaproteobacteria | 4472 | *Pectobacterium_atrosepticum_SCRI1043_uid57957* |
| Gammaproteobacteria | 3754 | *Cellvibrio_japonicus_Ueda107_uid59139* |
| Gammaproteobacteria | 3645 | *Shewanella_amazonensis_SB2B_uid58257* |
| Gammaproteobacteria | 4050 | *Yersinia_enterocolitica_8081_uid57741* |
| Gammaproteobacteria | 2407 | *Halorhodospira_halophila_SL1_uid58473* |
| Gammaproteobacteria | 1886 | *Moraxella_catarrhalis_RH4_uid48809* |
| Gammaproteobacteria | 4759 | *Escherichia_coli_55989_uid59383* |
| Gammaproteobacteria | 3474 | *Halomonas_elongata_DSM_2581_uid52781* |
| Gammaproteobacteria | 4619 | *Escherichia_coli_536_uid58531* |
| Gammaproteobacteria | 4525 | *Salmonella_enterica_serovar_Typhimurium_LT2_uid57799* |
| Gammaproteobacteria | 4557 | *Shigella_boydii_CDC_3083_94_uid58415* |
| Gammaproteobacteria | 3017 | *Nitrosococcus_oceani_ATCC_19707_uid58403* |
| Gammaproteobacteria | 4942 | *Serratia_proteamaculans_568_uid58725* |
| Gammaproteobacteria | 4237 | *Pantoea_ananatis_LMG_20103_uid46807* |
| Gammaproteobacteria | 3220 | *Allochromatium_vinosum_DSM_180_uid46083* |
| Gammaproteobacteria | 3565 | *Erwinia_amylovora_ATCC_49946_uid46943* |
| Gammaproteobacteria | 4399 | *Enterobacter_cloacae_SCF1_uid59969* |
| Gammaproteobacteria | 1866 | *Coxiella_burnetii_CbuG_Q212_uid58893* |
| Gammaproteobacteria | 5008 | *Citrobacter_koseri_ATCC_BAA_895_uid58143* |
| Gammaproteobacteria | 2832 | *Xylella_fastidiosa_9a5c_uid57849* |
| Gammaproteobacteria | 5925 | *Pseudomonas_aeruginosa_LESB58_uid59275* |
| Gammaproteobacteria | 3589 | *Edwardsiella_tarda_EIB202* |
| Gammaproteobacteria | 4271 | *Xanthomonas_campestris_8004_uid57595* |
| Spirochetes | 2809 | *Brachyspira_murdochii_DSM_12563_uid48819* |
| Spirochetes | 3702 | *Leptospira_interrogans_serovar_Lai_56601_uid57881* |
| Spirochetes | 2767 | *Treponema_denticola_ATCC_35405_uid57583* |
| Spirochetes | 4219 | *Spirochaeta_smaragdinae_DSM_11293_uid51369* |
| Spirochetes | 1036 | *Treponema_pallidum_Nichols_uid57585* |
| Tenericute | 646 | *Ureaplasma_urealyticum_serovar_10_ATCC_33699_uid59011* |
| Tenericute | 475 | *Mycoplasma_genitalium_G37_uid57707* |

**Tables S5: Statistical test for charged amino acid compositions in prokaryotic and eukaryotic genomes.**

|  | **Prokaryotic genomes (1051)** | | | **Eukaryotic genomes (12)** | | | ***P* value（Mann-Whitney U Test）** |
| --- | --- | --- | --- | --- | --- | --- | --- |
|  | **Mean** | **Median** | **Std. Deviation** | **Mean** | **Median** | **Std. Deviation** |  |
| [Acidic AA] | 0.116158 | 0.114663 | 0.011665 | 0.114955653 | 0.114169 | 0.003356858 | 0.950987 |
| [Basic AA] | 0.134245 | 0.132736 | 0.011495 | 0.142665842 | 0.14293502 | 0.002040538 | 0.000300118 |

**Tables S6: Sequences amount of ten kinds of human subcellular proteomes and their corresponding prokaryotic analogs used in evolutionary analysis. 1: Cytoplasm; 2: Cytoskeleton; 3: Endoplasmic reticulum; 4: Golgi; 5: Membrane; 6: Mitochondrion; 7: Nucleus; 8: Peroxisome; 9: Secretory; 10: Transmembrane.**

| **Subcellular Locations(type)** | 1 | 2 | 3 | 4 | 5 | 6 | 7 | 8 | 9 | 10 |
| --- | --- | --- | --- | --- | --- | --- | --- | --- | --- | --- |
| Human subcellular proteomes | 234 | 14 | 80 | 32 | 80 | 183 | 195 | 21 | 57 | 236 |
| Prokaryotic analogs | 6721 | 190 | 839 | 398 | 2055 | 10899 | 4257 | 926 | 557 | 2867 |

**Tables S7: Statistical test for acid AA content of various subcellular proteomes and their prokaryotic analogs.**

|  | **Prokaryotic analogs** | | **Eukaryotic subcellular proteomes** | | ***P* value （Mann-Whitney U Test）** |
| --- | --- | --- | --- | --- | --- |
|  | **Mean** | **Std. Deviation** | **Mean** | **Std. Deviation** |  |
| Cytoplasm | 0.131198 | 0.026340937 | 0.126356 | 0.02294295 | 0.145080712 |
| Cytoskeleton | 0.134831 | 0.026371649 | 0.143772 | 0.030295818 | 0.064039291 |
| ER | 0.111856 | 0.034250747 | 0.106884 | 0.031274422 | 0.344725976 |
| Golgi | 0.110829 | 0.031316817 | 0.114445 | 0.026512185 | 0.061498542 |
| Membrane | 0.128694 | 0.021941507 | 0.119035 | 0.024299089 | 0.023289993 |
| Mitochondria | 0.126895 | 0.032171142 | 0.104885 | 0.023427735 | 8.16486E-25 |
| Nucleus | 0.135879 | 0.028918983 | 0.130429 | 0.027729198 | 0.000728657 |
| Peroxisome | 0.119753 | 0.021780088 | 0.105916 | 0.012873122 | 0.045655454 |
| Extracellular | 0.110485 | 0.030110321 | 0.108825 | 0.025001946 | 0.264292999 |
| Transmembrane | 0.08225 | 0.038734188 | 0.090252 | 0.02411672 | 5.72798E-12 |

**Tables S8: Statistical test for basic AA content of various subcellular proteomes and their prokaryotic analogs.**

|  | **Prokaryotic analogs** | | **Eukaryotic subcellular proteomes** | | ***P* value**  **(Mann-Whitney U Test)** |
| --- | --- | --- | --- | --- | --- |
|  | **Mean** | **Std.**  **Deviation** | **Mean** | **Std. Deviation** |  |
| Cytoplasm | 0.129395 | 0.022570698 | 0.143127 | 0.024310773 | 7.00565E-15 |
| Cytoskeleton | 0.127618 | 0.018712787 | 0.151564 | 0.022228595 | 0.025842854 |
| ER | 0.124628 | 0.024438434 | 0.133773 | 0.020804641 | 0.431521271 |
| Golgi | 0.124335 | 0.028472217 | 0.151301 | 0.02282238 | 0.002164895 |
| Membrane | 0.129732 | 0.018749134 | 0.143544 | 0.02094474 | 2.85234E-06 |
| Mitochondria | 0.135436 | 0.035777397 | 0.146285 | 0.026290152 | 4.1136E-18 |
| Nucleus | 0.140166 | 0.02318812 | 0.151067 | 0.030953914 | 1.22754E-06 |
| Peroxisome | 0.123711 | 0.018418511 | 0.135293 | 0.017074657 | 0.024969877 |
| Extracellular | 0.111057 | 0.024376976 | 0.13026 | 0.023472866 | 2.97715E-05 |
| Transmembrane | 0.101852 | 0.031553527 | 0.11536 | 0.027683472 | 1.20007E-13 |

**Tables S9: Charge characteristic of different cytoskeleton protein families from Orthology (KO) System in KEGG.**

| **Category** | **Ortholog** | **KO** | **KEGG pathway** | **Name in Pathway** | **Mean Acidic AA frequency** | **Mean Basic AA frequency** |
| --- | --- | --- | --- | --- | --- | --- |
| Actin and actin binding proteins | ADF | K05765 | ko04810 | CFN | 0.154432 | 0.163204 |
|  | Arp23 | K05754-8 | ko04810 | Arp23 | 0.121583 | 0.147052 |
|  | Capping | K10364-5 | Ko04144 | CAPZB/A | 0.141373 | 0.139859 |
|  | Crosslink | K05699, K06114-5, K10366,K04437 | ko04810 | ACTN | 0.157213 | 0.151116 |
|  | Formins | K02184, K10367 | ko04320 | Capu（FMNs） | 0.126284 | 0.133757 |
|  | Gelsolin | K05761,K05768,K08007, K08017, K10368-9 | ko04810 | GSN,ERM | 0.142421 | 0.148096 |
|  | Myosins | K10356-7,9, K10360-2 K10351-2 K05738, K08834 | ko04810 | MLC | 0.15817 | 0.162174 |
|  | Profilin | K05759 | ko04810 | PFN | 0.099003 | 0.122551 |
|  | actins | K10354-5, K05692 | ko04810 | F-actin | 0.128653 | 0.123783 |
|  | MreB | K03569 | No data | No data | 0.129247 | 0.121384 |
|  | Tropomodulin | K10370 | No data | No data | 0.17107 | 0.139317 |
|  | Tropomyosins | K10373-5, K09290, | Ko5410 | TPM | 0.257855 | 0.190914 |
|  | Troponins | K05865, K10371 | Ko5410 | TnC | 0.224393 | 0.175264 |
|  | Wiskott | K05747 | ko04810 | NWASP | 0.109705 | 0.130152 |
| Intermediate filaments  and binding proteins | Interbind | K06114-5, K10380-8 K04437, K04985 | Ko05205 | Ankyrin/Filamin | 0.145425 | 0.149025 |
|  | InterIandII | K07604-5 | No data | No data | 0.136555 | 0.124537 |
|  | InterIII | K05640, k07606-7, k07610 | Ko04630 | GFAP | 0.151137 | 0.147421 |
|  | InterIV | K07608-9, K10376-7, K04572-4 | K05014 | SOD1,NEFL,NEFM(binding),NEFH,ALS2 | 0.211115 | 0.160055 |
|  | InterO | K10378-9 | No data | No data | 0.157186 | 0.158817 |
|  | InterV | K07611 | Ko04214 | Lamin | 0.177632 | 0.173451 |
| Microtubules and binding proteins | CLIPs | K10421-3 | Ko04150 | CLIP-170 | 0.13915 | 0.154116 |
|  | Dynactins | K10424-8, K04648 | K04962 | Dynactin | 0.12466 | 0.135967 |
|  | Dyneins | K10408-20, K11143 | K04962 | Dyneins | 0.138095 | 0.144336 |
|  | DyneinsAPC | K02085 | ko04810 | APC | 0.122046 | 0.144482 |
|  | Kinesins | K10400-7, K10392-9 | Ko04144 | KIF5 | 0.14111 | 0.159585 |
|  | MAPs | K10429-36,  K04380 | Ko05010 | Tau,1/4bingding | 0.141086 | 0.154202 |
|  | Stathmin | K04381 | Ko04010 | STMN1 | 0.230886 | 0.235144 |
|  | Microtubules | K07374-5, K10389-91 | Ko05130 | TUBA/B | 0.132626 | 0.113774 |
|  | FtsZ | K03531 | Ko04112 | No data | 0.135026 | 0.105729 |
|  | MinD | K03609 | No data | No data | 0.142835 | 0.134070 |
|  | ParA | K03496 | No data | No data | 0.118401 | 0.118867 |
|  | ParB | K03497 | No data | No data | 0.146611 | 0.161087 |

**Table S11: Theoretical isoelectric point (PI) of two proteins in each protein-protein interaction pair for human cytoskeleton.**

| **Protein A** | **PI** | **Protein B** | **PI** |
| --- | --- | --- | --- |
| NP_060120.2 | 4.707 | NP_001605.1 | 5.077 |
| NP_006861.1 | 7.645 | NP_001605.1 | 5.077 |
| NP_001447.2 | 5.607 | NP_001655.1 | 5.684 |
| NP_891556.1 | 5.256 | NP_001655.1 | 5.684 |
| AAL96658.1 | 6.867 | NP_001655.1 | 5.684 |
| AAH11604.1 | 4.972 | NP_001655.1 | 5.684 |
| NP_891992.1 | 6.464 | NP_001655.1 | 5.684 |
| NP_443156.2 | 7.185 | NP_001655.1 | 5.684 |
| NP_003371.2 | 4.746 | NP_079120.1 | 5.151 |
| NP_057225.1 | 6.118 | NP_079120.1 | 5.151 |
| NP_001005360.1 | 7.094 | NP_005013.1 | 8.163 |
| NP_003932.3 | 7.73 | NP_005013.1 | 8.163 |
| NP_001008493.1 | 6.513 | NP_005013.1 | 8.163 |
| NP_061916.3 | 5.182 | NP_005013.1 | 8.163 |
| NP_573571.1 | 11.425 | NP_005013.1 | 8.163 |
| NP_006781.1 | 9.312 | NP_001449.3 | 5.537 |
| NP_665696.1 | 7.186 | NP_001449.3 | 5.537 |
| NP_000263.2 | 4.813 | NP_001449.3 | 5.537 |
| NP_009055.2 | 4.93 | NP_001094.1 | 5.057 |
| NP_001095.1 | 5.163 | NP_001094.1 | 5.057 |
| NP_958782.1 | 5.556 | NP_003118.1 | 4.95 |
| NP_958782.1 | 5.556 | NP_003117.2 | 4.657 |
| NP_008877.1 | 5.688 | NP_003117.2 | 4.657 |
| NP_932326.2 | 9.672 | NP_006280.2 | 5.624 |
| NP_001093.1 | 5.006 | NP_054706.1 | 5.258 |
| NP_932326.2 | 9.672 | NP_001095.1 | 5.163 |
| NP_932326.2 | 9.672 | NP_006054.2 | 4.875 |
| NP_115967.1 | 6.378 | NP_001094.1 | 5.057 |
| NP_597709.2 | 9.15 | NP_001094.1 | 5.057 |
| NP_003274.2 | 5.675 | NP_000357.3 | 4.381 |
| NP_055363.1 | 4.897 | NP_000357.3 | 4.381 |
| NP_003280.2 | 4.333 | NP_000357.3 | 4.381 |
| NP_001290.2 | 9.51 | NP_689476.2 | 4.353 |
| NP_055363.1 | 4.897 | NP_689476.2 | 4.353 |
| NP_002364.5 | 4.534 | NP_005991.1 | 4.661 |
| NP_852610.1 | 7.893 | NP_005991.1 | 4.661 |
| NP_036369.2 | 4.96 | NP_005991.1 | 4.661 |
| NP_002364.5 | 4.534 | NP_001060.1 | 4.494 |
| NP_054890.1 | 9.916 | NP_001060.1 | 4.494 |
| NP_733832.1 | 8.99 | NP_001060.1 | 4.494 |
| NP_000263.2 | 4.813 | NP_001060.1 | 4.494 |
| NP_060654.1 | 8.326 | NP_001060.1 | 4.494 |
| NP_149052.1 | 9.658 | NP_001060.1 | 4.494 |
| NP_733832.1 | 8.99 | NP_001061.2 | 5.696 |
| NP_006313.1 | 7.908 | NP_001061.2 | 5.696 |
| NP_001894.2 | 5.856 | NP_001093.1 | 5.006 |
| NP_958782.1 | 5.556 | NP_003371.2 | 4.746 |
| NP_932326.2 | 9.672 | NP_054706.1 | 5.258 |
| NP_006082.3 | 8.091 | NP_054706.1 | 5.258 |
| NP_001030126.1 | 6.408 | NP_054706.1 | 5.258 |
| NP_597709.2 | 9.15 | NP_054706.1 | 5.258 |
| NP_001448.2 | 5.303 | NP_001447.2 | 5.607 |
| NP_006781.1 | 9.312 | NP_001447.2 | 5.607 |
| NP_000263.2 | 4.813 | NP_001447.2 | 5.607 |
| NP_001019386.1 | 5.994 | NP_001447.2 | 5.607 |
| NP_115967.1 | 6.378 | NP_001095.1 | 5.163 |
| NP_006413.2 | 5.746 | NP_003877.2 | 6.547 |
| NP_003361.1 | 9.279 | NP_000368.1 | 6.191 |
| NP_003378.3 | 11.957 | NP_000368.1 | 6.191 |
| NP_005711.1 | 8.121 | NP_000368.1 | 6.191 |
| NP_573571.1 | 11.425 | NP_000368.1 | 6.191 |
| NP_958782.1 | 5.556 | NP_002435.1 | 5.981 |
| NP_006781.1 | 9.312 | NP_001093.1 | 5.006 |
| NP_852610.1 | 7.893 | NP_002364.5 | 4.534 |
| NP_003940.3 | 4.343 | AAA60120.1 | 4.679 |
| NP_006022.3 | 5.161 | AAA60120.1 | 4.679 |
| NP_003467.1 | 8.321 | NP_001093.1 | 5.006 |
| NP_006384.1 | 7.975 | NP_001093.1 | 5.006 |
| NP_597709.2 | 9.15 | NP_001093.1 | 5.006 |
| NP_061916.3 | 5.182 | NP_003361.1 | 9.279 |
| NP_004073.2 | 5.412 | NP_899236.1 | 5.311 |
| NP_005392.2 | 8.169 | NP_004380.2 | 5.54 |
| NP_003932.3 | 7.73 | NP_003378.3 | 11.957 |
| NP_003572.2 | 6.531 | NP_004399.2 | 6.757 |
| NP_036441.1 | 5.199 | NP_001005360.1 | 7.094 |
| NP_001008709.1 | 6.196 | NP_003479.1 | 4.549 |
| NP_001377.1 | 5.934 | NP_001060.1 | 4.494 |
| NP_003616.2 | 5.688 | NP_003613.2 | 5.268 |
| NP_003651.1 | 5.289 | NP_003613.2 | 5.268 |
| NP_003617.1 | 5.854 | NP_003613.2 | 5.268 |
| NP_003616.2 | 5.688 | NP_003612.1 | 5.778 |
| NP_003651.1 | 5.289 | NP_003612.1 | 5.778 |
| NP_003617.1 | 5.854 | NP_003612.1 | 5.778 |
| NP_003651.1 | 5.289 | NP_003616.2 | 5.688 |
| NP_056391.1 | 6.543 | NP_003616.2 | 5.688 |
| NP_003617.1 | 5.854 | NP_003616.2 | 5.688 |
| NP_056391.1 | 6.543 | NP_003651.1 | 5.289 |
| NP_003617.1 | 5.854 | NP_003651.1 | 5.289 |
| NP_000263.2 | 4.813 | NP_001448.2 | 5.303 |
| NP_001019386.1 | 5.994 | NP_001448.2 | 5.303 |
| NP_008921.1 | 5.116 | NP_001005386.1 | 6.808 |
| NP_006637.2 | 5.985 | NP_005712.1 | 5.466 |
| NP_008921.1 | 5.116 | NP_005712.1 | 5.466 |
| NP_005710.1 | 8.621 | NP_005712.1 | 5.466 |
| NP_005712.1 | 5.466 | NP_005708.1 | 5.249 |
| NP_004915.2 | 5.037 | NP_001095.1 | 5.163 |
| NP_573571.1 | 11.425 | NP_003572.2 | 6.531 |
| NP_005727.1 | 6.198 | NP_008877.1 | 5.688 |
| NP_004073.2 | 5.412 | NP_008877.1 | 5.688 |
| NP_001020119.1 | 5.748 | NP_003932.3 | 7.73 |
| AAH11604.1 | 4.972 | NP_733832.1 | 8.99 |
| NP_006313.1 | 7.908 | NP_057521.1 | 5.366 |
| NP_005710.1 | 8.621 | NP_008921.1 | 5.116 |
| NP_006313.1 | 7.908 | NP_006022.3 | 5.161 |
| NP_006650.1 | 6.405 | NP_006022.3 | 5.161 |
| NP_004073.2 | 5.412 | NP_009165.1 | 5.078 |
| NP_001019386.1 | 5.994 | NP_006823.1 | 6.26 |
| NP_055912.1 | 8.082 | NP_003379.3 | 6.227 |
| NP_054706.1 | 5.258 | NP_001894.2 | 5.856 |
| NP_037394.2 | 6.851 | NP_291024.1 | 4.429 |
| NP_115967.1 | 6.378 | NP_006384.1 | 7.975 |
| NP_003280.2 | 4.333 | NP_005442.2 | 8.176 |
| NP_003617.1 | 5.854 | NP_056391.1 | 6.543 |
| NP_443156.2 | 7.185 | AAH11604.1 | 4.972 |
| NP_055083.1 | 5.159 | NP_055912.1 | 8.082 |
| NP_001008709.1 | 6.196 | NP_055826.1 | 6.378 |
| NP_060138.1 | 4.77 | NP_001061.2 | 5.696 |
| NP_003370.2 | 5.843 | NP_005150.1 | 4.987 |
| NP_060120.2 | 4.707 | NP_001008709.1 | 6.196 |
| NP_690601.1 | 6.978 | NP_005709.1 | 8.422 |
| NP_005709.1 | 8.422 | NP_005708.1 | 5.249 |
| NP_005709.1 | 8.422 | NP_005710.1 | 8.621 |
| NP_056391.1 | 6.543 | NP_055325.2 | 5.884 |
| NP_001447.2 | 5.607 | NP_001782.1 | 6.139 |
| NP_001782.1 | 6.139 | NP_000368.1 | 6.191 |
| NP_001005386.1 | 6.808 | NP_005708.1 | 5.249 |
| NP_005712.1 | 5.466 | NP_690601.1 | 6.978 |
| NP_891556.1 | 5.256 | NP_001782.1 | 6.139 |
| NP_061916.3 | 5.182 | NP_057421.1 | 9.198 |
| NP_005711.1 | 8.121 | NP_005709.1 | 8.422 |
| NP_005711.1 | 8.121 | NP_005708.1 | 5.249 |
| NP_005712.1 | 5.466 | NP_001782.1 | 6.139 |
| NP_003932.3 | 7.73 | NP_001782.1 | 6.139 |
| NP_005875.1 | 10.244 | NP_001782.1 | 6.139 |
| NP_008921.1 | 5.116 | NP_001782.1 | 6.139 |
| NP_061821.1 | 7.889 | NP_001782.1 | 6.139 |
| NP_056097.1 | 9.384 | NP_036458.2 | 5.127 |
| NP_891992.1 | 6.464 | NP_001782.1 | 6.139 |
| NP_006280.2 | 5.624 | NP_054706.1 | 5.258 |
| NP_000368.1 | 6.191 | NP_005150.1 | 4.987 |
| NP_899236.1 | 5.311 | NP_000517.2 | 4.78 |
| NP_003266.1 | 4.715 | NP_689476.2 | 4.353 |
| NP_001139.3 | 4.74 | NP_003119.2 | 5.164 |
| NP_001930.2 | 5.789 | NP_009055.2 | 4.93 |
| NP_002304.3 | 8.414 | NP_001605.1 | 5.077 |
| NP_066926.1 | 5 | NP_003266.1 | 4.715 |
| NP_001605.1 | 5.077 | NP_005498.1 | 7.981 |
| NP_001605.1 | 5.077 | NP_068733.1 | 7.859 |
| NP_001605.1 | 5.077 | NP_852133.1 | 4.286 |
| NP_001092.1 | 5.069 | NP_005498.1 | 7.981 |
| NP_001092.1 | 5.069 | NP_068733.1 | 7.859 |
| NP_001092.1 | 5.069 | NP_006861.1 | 7.645 |
| NP_001782.1 | 6.139 | NP_689449.1 | 6.73 |
| NP_002267.2 | 4.752 | AAA60120.1 | 4.679 |
| NP_002266.2 | 4.392 | AAA60120.1 | 4.679 |
| NP_002266.2 | 4.392 | NP_002304.3 | 8.414 |
| NP_002266.2 | 4.392 | NP_056008.1 | 5.23 |
| NP_002266.2 | 4.392 | NP_443156.2 | 7.185 |
| NP_958782.1 | 5.556 | NP_005150.1 | 4.987 |
| NP_003274.2 | 5.675 | NP_003617.1 | 5.854 |
| NP_003371.2 | 4.746 | NP_002304.3 | 8.414 |
| NP_006748.1 | 5.951 | NP_003940.3 | 4.343 |
| NP_005498.1 | 7.981 | NP_005150.1 | 4.987 |
| NP_002304.3 | 8.414 | NP_037444.1 | 5.051 |
| NP_003612.1 | 5.778 | NP_068707.1 | 7.64 |
| NP_689449.1 | 6.73 | NP_114060.1 | 7.58 |
| NP_114165.1 | 5.277 | NP_114060.1 | 7.58 |
| NP_036458.2 | 5.127 | NP_055083.1 | 5.159 |
| NP_003378.3 | 11.957 | NP_005150.1 | 4.987 |
| NP_006000.2 | 4.666 | NP_055413.1 | 7.806 |
| AAH07560.1 | 8.449 | NP_005150.1 | 4.987 |
| NP_003117.2 | 4.657 | NP_003361.1 | 9.279 |
| NP_003361.1 | 9.279 | NP_005013.1 | 8.163 |
| NP_003361.1 | 9.279 | NP_054706.1 | 5.258 |
| NP_056303.3 | 9.414 | NP_291024.1 | 4.429 |
| NP_001655.1 | 5.684 | NP_005210.3 | 5.025 |
| NP_001779.3 | 8.822 | AAF23374.1 | 9.363 |
| NP_002897.1 | 5.945 | NP_003370.2 | 5.843 |
| NP_002435.1 | 5.981 | NP_003370.2 | 5.843 |
| NP_000259.1 | 6.04 | NP_003370.2 | 5.843 |
| NP_066932.1 | 4.703 | NP_001604.1 | 4.99 |
| NP_005498.1 | 7.981 | NP_001604.1 | 4.99 |
| NP_003371.2 | 4.746 | NP_001034230.1 | 8.243 |
| NP_003370.2 | 5.843 | NP_001092.1 | 5.069 |
| NP_067651.2 | 8.733 | NP_005150.1 | 4.987 |
| NP_005900.1 | 4.417 | NP_001092.1 | 5.069 |
| AAH66644.1 | 5.048 | NP_001008709.1 | 6.196 |
| NP_612410.1 | 9.063 | NP_114098.1 | 6.187 |
| NP_733832.1 | 8.99 | NP_005900.1 | 4.417 |
| NP_055413.1 | 7.806 | NP_005766.2 | 9.934 |
| NP_001782.1 | 6.139 | NP_064624.1 | 10.519 |
| NP_005013.1 | 8.163 | NP_001092.1 | 5.069 |
| NP_892006.1 | 5.129 | NP_005150.1 | 4.987 |
| NP_543157.1 | 4.148 | NP_005013.1 | 8.163 |
| NP_055949.2 | 5.956 | NP_001655.1 | 5.684 |
| NP_068707.1 | 7.64 | NP_009055.2 | 4.93 |
| NP_000357.3 | 4.381 | NP_001092.1 | 5.069 |
| NP_733832.1 | 8.99 | NP_060644.4 | 6.905 |
| NP_689476.2 | 4.353 | NP_001092.1 | 5.069 |
| NP_057421.1 | 9.198 | NP_000368.1 | 6.191 |
| NP_057421.1 | 9.198 | NP_003932.3 | 7.73 |
| NP_000368.1 | 6.191 | NP_001008493.1 | 6.513 |
| NP_003932.3 | 7.73 | NP_573571.1 | 11.425 |
| NP_003932.3 | 7.73 | NP_003361.1 | 9.279 |
| NP_005222.2 | 4.99 | NP_003950.1 | 6.228 |
| NP_066932.1 | 4.703 | NP_001092.1 | 5.069 |
| NP_002009.1 | 5.625 | NP_001092.1 | 5.069 |
| NP_001020276.1 | 10.087 | NP_001092.1 | 5.069 |
| NP_057332.1 | 4.99 | NP_001008709.1 | 6.196 |
| NP_000546.2 | 9.178 | NP_001008709.1 | 6.196 |
| NP_004359.1 | 7.009 | NP_001092.1 | 5.069 |
| NP_002813.2 | 8.571 | NP_005150.1 | 4.987 |
| NP_036266.2 | 9.862 | NP_001092.1 | 5.069 |
| NP_932326.2 | 9.672 | NP_001092.1 | 5.069 |
| AAH07560.1 | 8.449 | NP_001092.1 | 5.069 |
| AAH21090.1 | 5.304 | NP_005150.1 | 4.987 |
| NP_009005.1 | 6.251 | NP_001092.1 | 5.069 |
| NP_006637.2 | 5.985 | NP_001092.1 | 5.069 |
| NP_631905.1 | 8.598 | NP_005150.1 | 4.987 |
| NP_008921.1 | 5.116 | NP_001092.1 | 5.069 |
| NP_005711.1 | 8.121 | NP_001092.1 | 5.069 |
| NP_006384.1 | 7.975 | NP_001092.1 | 5.069 |
| NP_938073.1 | 7.381 | NP_005150.1 | 4.987 |
| NP_631905.1 | 8.598 | NP_001092.1 | 5.069 |
| NP_543151.1 | 8.184 | NP_005150.1 | 4.987 |
| NP_008990.2 | 4.862 | NP_005150.1 | 4.987 |
| NP_054706.1 | 5.258 | NP_000168.1 | 5.834 |
| NP_003361.1 | 9.279 | NP_000168.1 | 5.834 |
| NP_005709.1 | 8.422 | NP_001005386.1 | 6.808 |
| NP_005711.1 | 8.121 | NP_001005386.1 | 6.808 |
| NP_005711.1 | 8.121 | NP_690601.1 | 6.978 |
| NP_005709.1 | 8.422 | NP_005712.1 | 5.466 |
| NP_009058.1 | 5.955 | NP_001605.1 | 5.077 |
| NP_066932.1 | 4.703 | NP_001605.1 | 5.077 |
| NP_004193.1 | 5.015 | NP_001605.1 | 5.077 |
| NP_002364.5 | 4.534 | NP_001605.1 | 5.077 |
| NP_958782.1 | 5.556 | NP_001605.1 | 5.077 |
| NP_003361.1 | 9.279 | NP_001605.1 | 5.077 |
| NP_112494.3 | 9.142 | NP_001008709.1 | 6.196 |
| NP_002364.5 | 4.534 | NP_005900.1 | 4.417 |
| NP_071324.1 | 5.427 | NP_005900.1 | 4.417 |
| NP_001060.1 | 4.494 | NP_002366.2 | 5.031 |
| NP_003266.1 | 4.715 | NP_004534.2 | 9.547 |
| NP_037373.2 | 5.953 | NP_001605.1 | 5.077 |
| AAH21090.1 | 5.304 | NP_001605.1 | 5.077 |
| NP_003378.3 | 11.957 | NP_005222.2 | 4.99 |
| NP_004399.2 | 6.757 | NP_005222.2 | 4.99 |
| NP_001005360.1 | 7.094 | NP_005222.2 | 4.99 |
| NP_036441.1 | 5.199 | NP_005222.2 | 4.99 |
| NP_005712.1 | 5.466 | NP_005222.2 | 4.99 |
| NP_003932.3 | 7.73 | NP_005222.2 | 4.99 |

**Table S12: Theoretical isoelectric point (PI) difference distribution of two proteins in each protein-protein interaction pair.**

|  | | Non-cytoskeleton PPIs | Cytoskeleton PPIs | Random non-interacting  cytoskeleton pairs |
| --- | --- | --- | --- | --- |
| Mean of (PImax-PImin) | | 1.618094 | 1.838424 | 1.798737 |
| Comparison of non-cytoskeleton and cytoskeleotn PPIs | Mann-Whitney Test, 1-tailed | 2.89E-05 | |  |
|  | Kolmogorov-Smirnov Test, 1-tailed | 3.12E-06 | |  |
| Comparison of cytoskeleotn PPIs and random non-interacting  cytoskeleton pairs | Mann-Whitney Test, 1-tailed |  | 0.067719 | |
|  | Kolmogorov-Smirnov Test, 1-tailed |  | 0.004352 | |

**Table S13: The Glu amino acid (AA) content of cytoskeleton PPIs and non cytoskeleton PPIs.**

| AA type | Mean content of  Cytoskeleton PPI | Mean content of  Cytoskeleton non PPI | P value | |
| --- | --- | --- | --- | --- |
|  |  |  | Mann-Whitney Test,  1-tailed | Kolmogorov-Smirnov Test,  1-tailed |
| Glu | 0.115 | 0.088 | 0.0327848 | 0.0061596 |

**Figures**

**Figure S1. The flow chart of sorting sequences into different subcellular locations.**

**
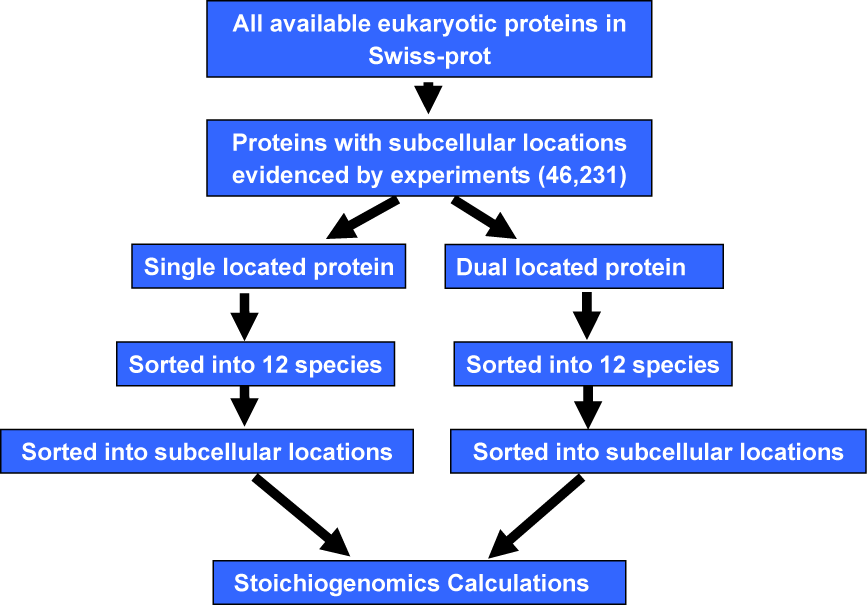
**

**Figure S2. Oxygen contents and its multiple variance analysis in various subcellular proteomes. (A)** Turkey's multiple comparisons of oxygen contents among different subcellular proteomes. **(B)** Boxplot showing the oxygen contents of various subcellular proteomes, calculated from single location proteins. [O]: oxygen content.

**
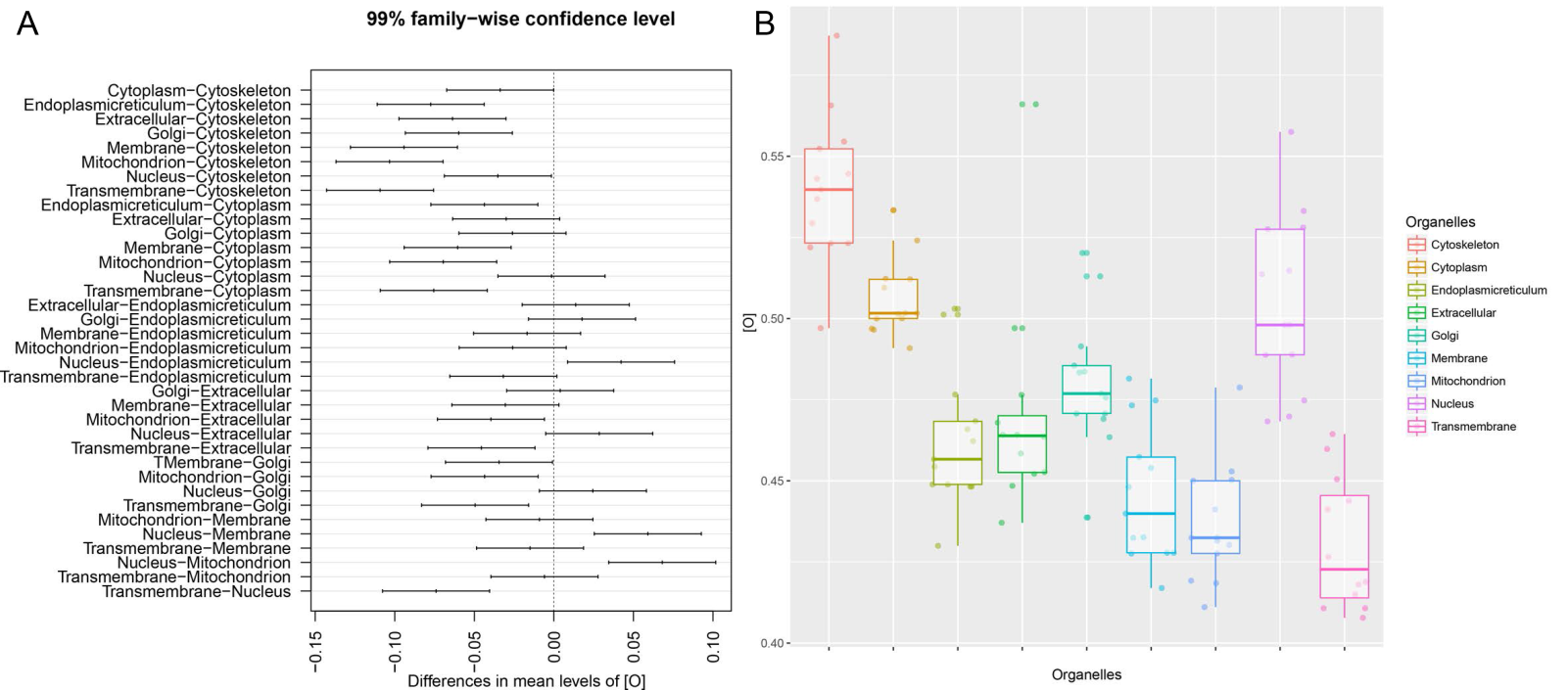
**

**Figure S3. Sulfur contents and its multiple variance analysis in various subcellular proteomes. (A)** Turkey's multiple comparisons of sulfur contents among different subcellular proteomes. **(B)** Boxplot showing the sulfur contents of various subcellular proteomes, calculated from single location proteins. [S]: sulfur content.

**
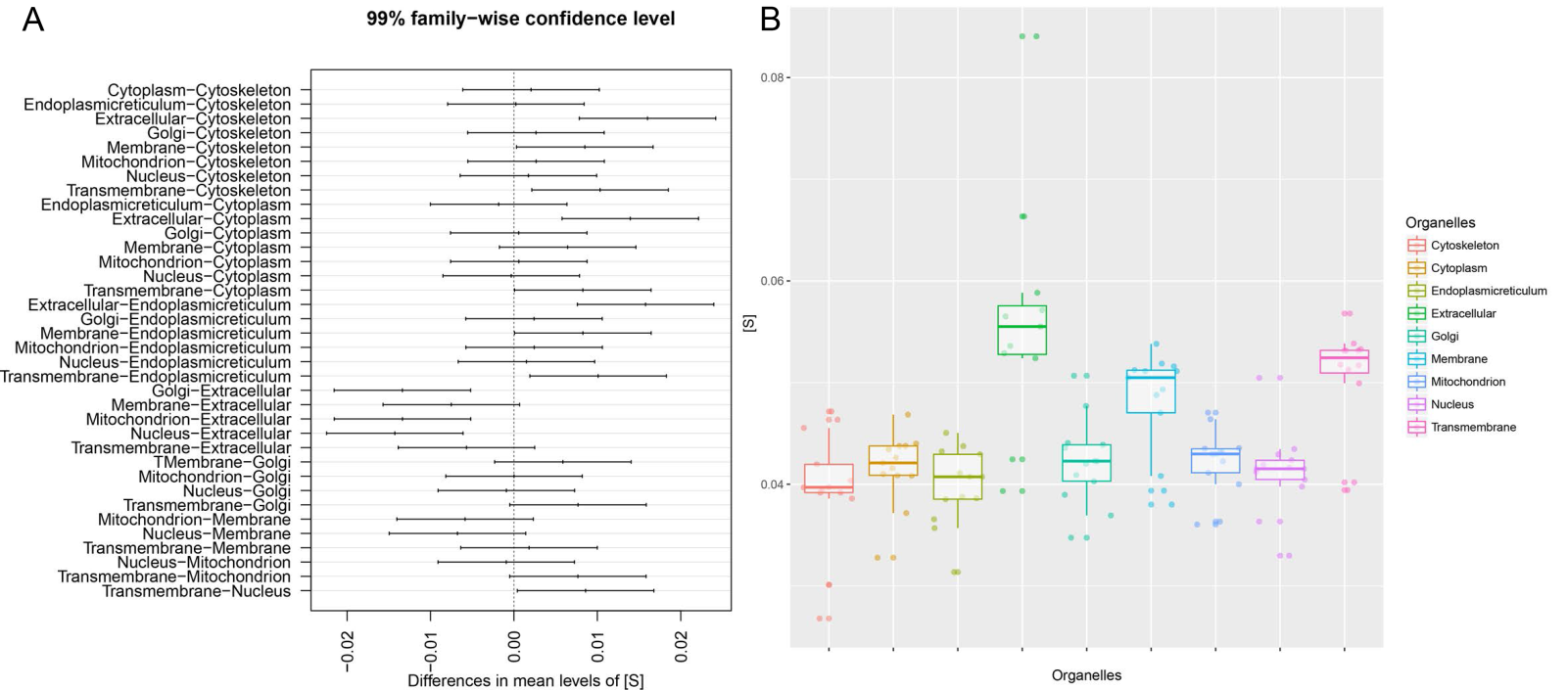
**

**Figure S4. Carbon contents and its multiple variance analysis in various subcellular proteomes. (A)** Turkey's multiple comparisons of carbon contents among different subcellular proteomes. **(B)** Boxplot showing the carbon contents of various subcellular proteomes calculated from single location proteins. [C]: carbon content.

**
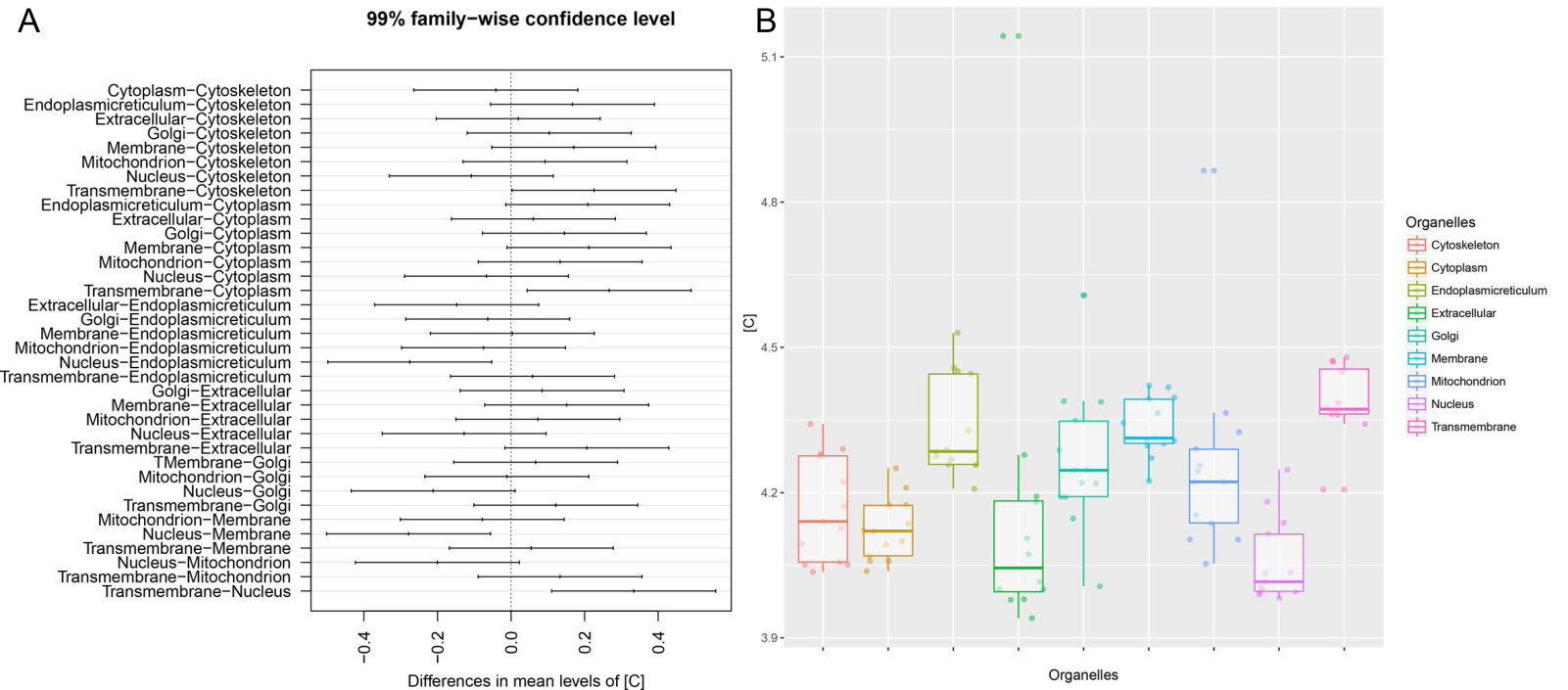
**

**Figure S5. Nitrogen contents and its multiple variance analysis in various subcellular proteomes.** Turkey's multiple comparisons of nitrogen contents among different subcellular proteomes. Red lines indicate that the nitrogen content of nucleus proteome has significant difference with all the other investigated subcellular proteomes at 99% confidence level.

**
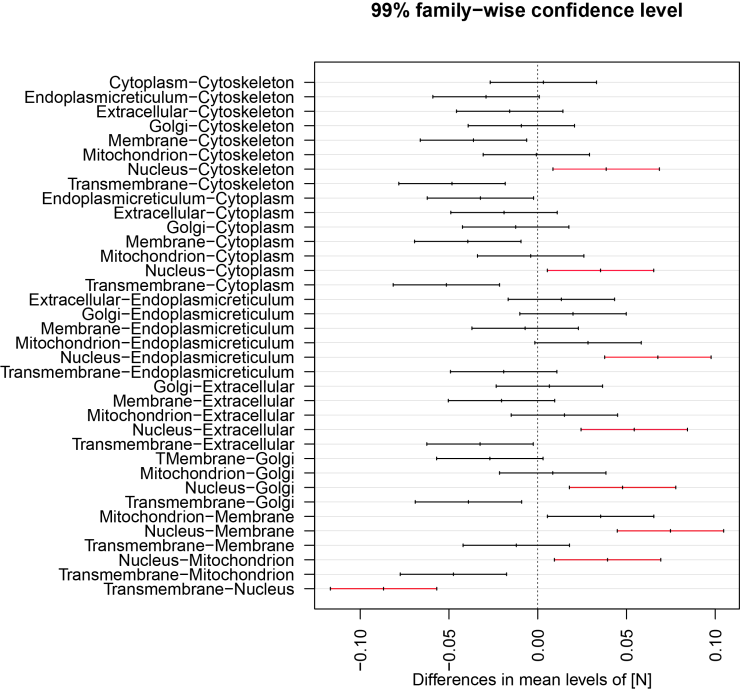
**

**Figure S6. Hydrogen contents and its multiple variance analysis in various subcellular proteomes.** Turkey's multiple comparisons of hydrogen contents among different subcellular proteomes. Red lines indicate that the hydrogen content of extracellular proteome has significant difference with all the other investigated subcellular proteomes at 99% confidence level.

**
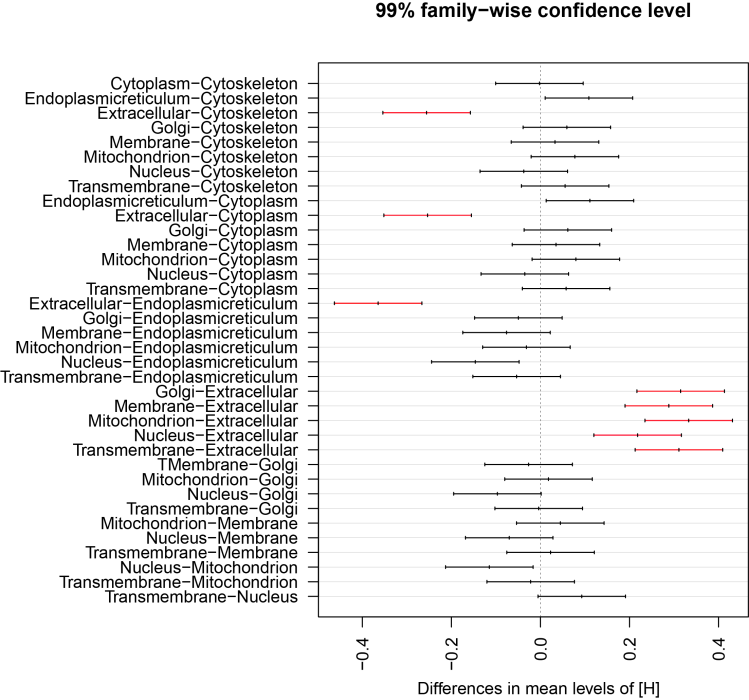
**

**Figure S7. Acidic amino acid contents and its multiple variance analysis in various subcellular proteomes.** **(A)** Turkey's multiple comparisons of acidic AA contents among different subcellular proteomes. Red lines indicate that acidic AA content of cytoskeleton is higher than all the other investigated subcellular proteomes. **(B)** Boxplot showing the acidic AA contents of various subcellular proteomes calculated from single location proteins. [Acidic]: Acidic amino acid contents.

**
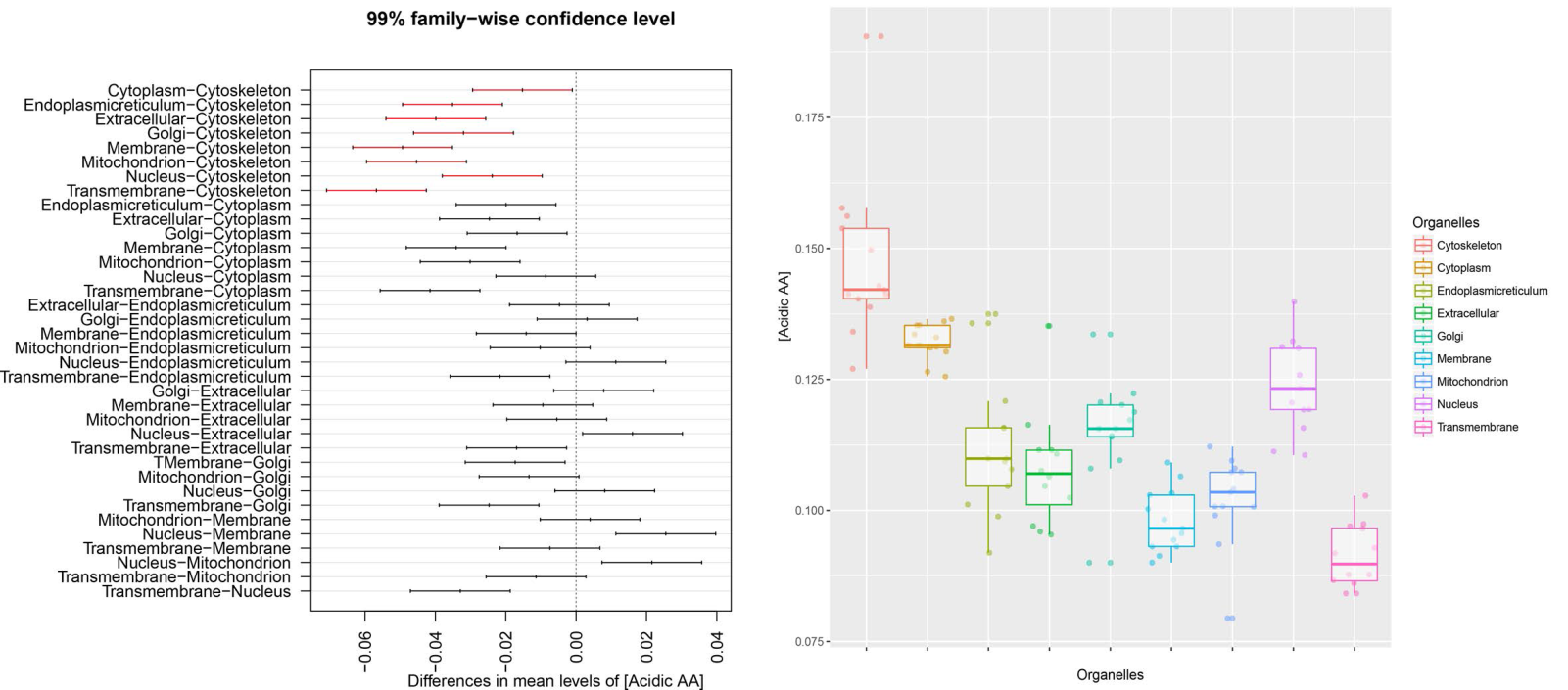
**

**Figure S8. Basic amino acid contents and its multiple variance analysis in various subcellular proteomes. (A)** Turkey's multiple comparisons of basic AA contents among different subcellular proteomes. **(B)** Boxplot showing the basic AA contents of various subcellular proteomes calculated from single location proteins. [Basic]: basic amino acid contents.

**
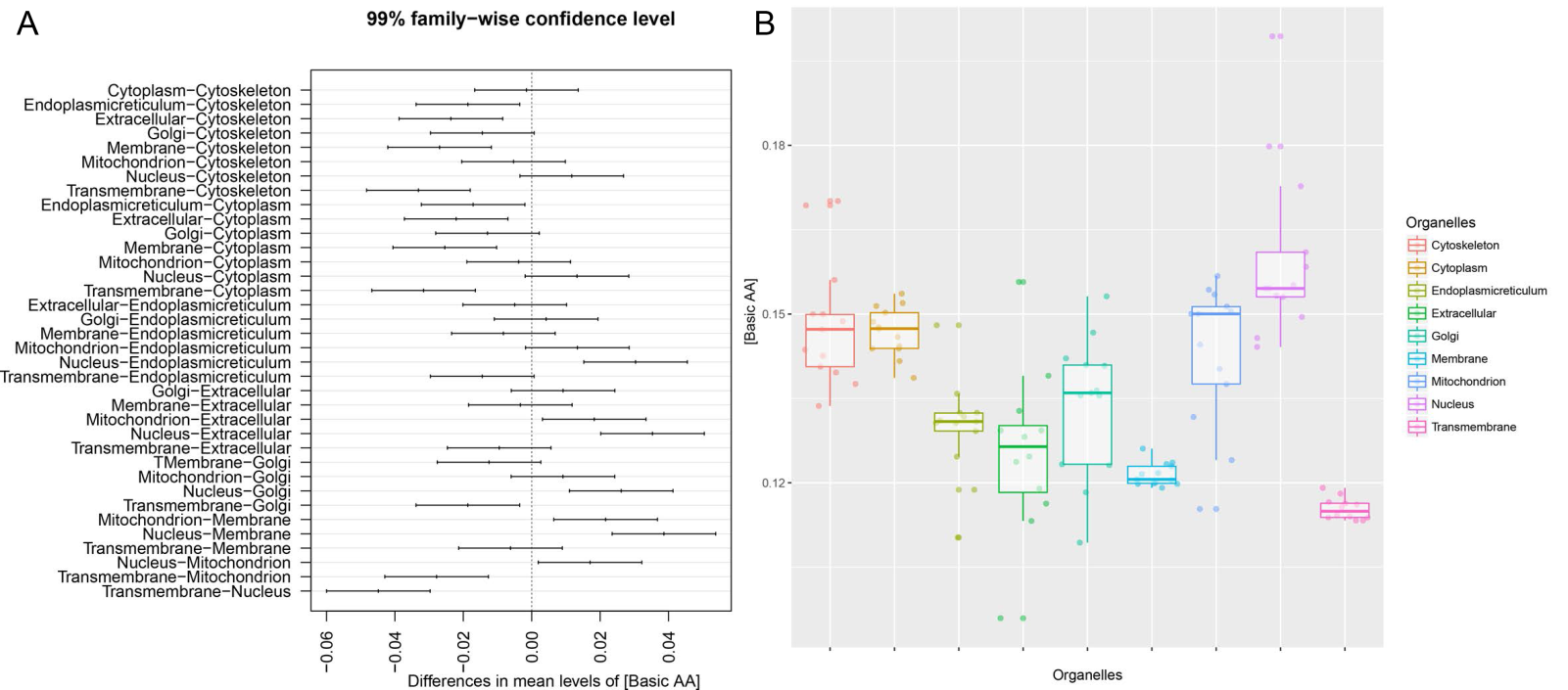
**

**Figure S9 Pairwise correlation between different stoichiogenomic characteristics. Only correlation values that larger than 0.8 were shown.**

**
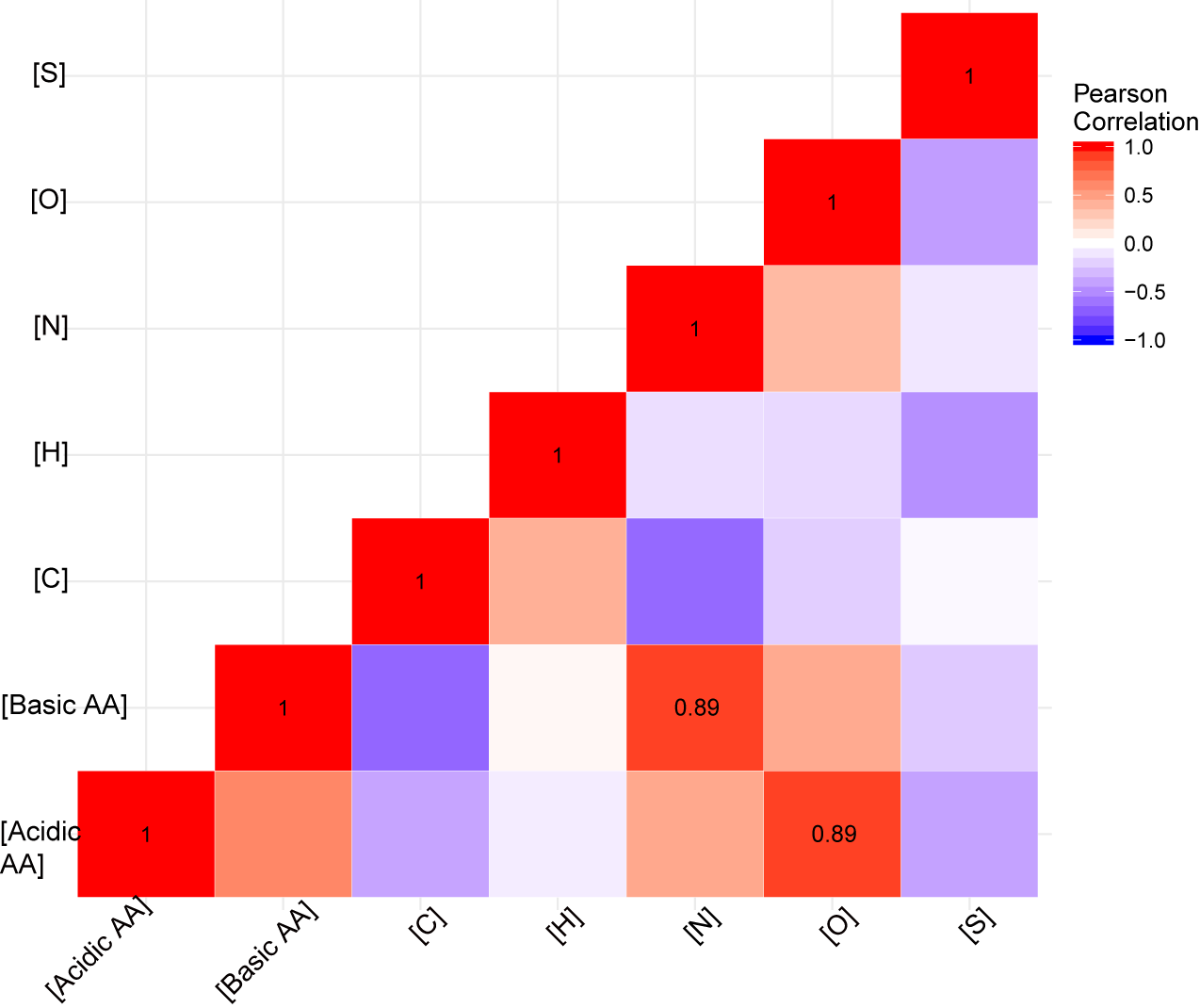
**

**Figure S10 The distribution of average value of charged amino acid frequencies of different subcellular proteomes in different species, calculated from dual located proteins. (A)** Acidic AA frequency. **(B)** Basic AA frequency.
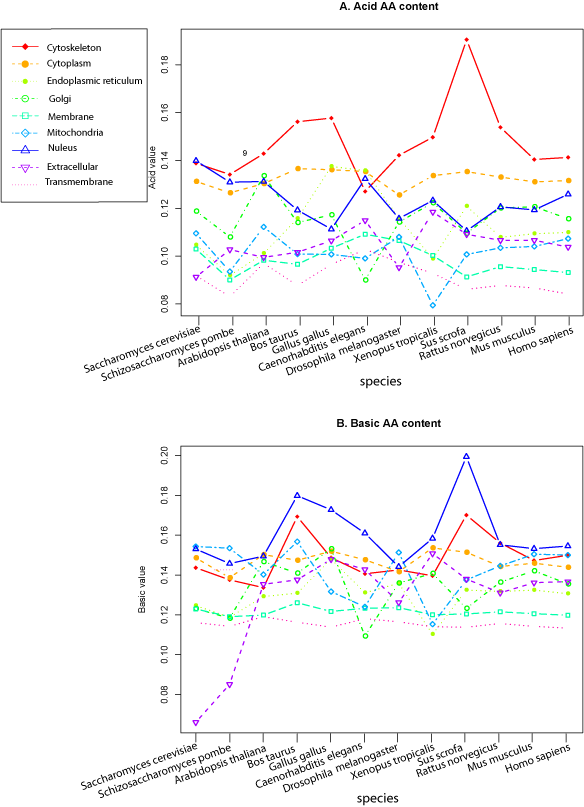

Supplement: Supplementary file 1 — Tables S1 to S9, S11 to S13 and Figures S1 to S10. Table S10: The protein-protein interaction (PPI) types between each cytoskeleton protein and its associated upstream and downstream proteins in KEGG pathway. (ZIP 2429 kb) [file 12864_2018_4845_MOESM1_ESM.zip › Additional files_20180516.docx]
